# Advances in Wireless, Batteryless, Implantable Electronics for Real-Time, Continuous Physiological Monitoring

**DOI:** 10.1007/s40820-023-01272-6

**Published:** 2023-12-15

**Authors:** Hyeonseok Kim, Bruno Rigo, Gabriella Wong, Yoon Jae Lee, Woon-Hong Yeo

**Affiliations:** 1 IEN Center for Wearable Intelligent Systems and Healthcare, Georgia Institute of Technology, Atlanta, GA 30332 USA; 2 George W. Woodruff School of Mechanical Engineering, Georgia Institute of Technology, Atlanta, GA 30332 USA; 3 School of Electrical and Computer Engineering, Georgia Institute of Technology, Atlanta, GA 30332 USA; 4 Wallace H. Coulter Department of Biomedical Engineering, Georgia Tech and Emory University School of Medicine, Atlanta, GA 30332 USA; 5 Parker H. Petit Institute for Bioengineering and Biosciences, Institute for Materials, Institute for Robotics and Intelligent Machines, Georgia Institute of Technology, Atlanta, GA 30332 USA

**Keywords:** Implantable electronics, Biomedical systems, Batteryless devices, Wireless electronics, Physiological signal monitoring

## Abstract

This review summarizes recent progress in developing wireless, batteryless, fully implantable biomedical devices for real-time continuous physiological signal monitoring, focusing on advancing human health care. Design considerations, such as biological constraints, energy sourcing, and wireless communication, are discussed in achieving the desired performance of the devices and enhanced interface with human tissues. In addition, we review the recent achievements in materials used for developing implantable systems, emphasizing their importance in achieving multi-functionalities, biocompatibility, and hemocompatibility. The wireless, batteryless devices offer minimally invasive device insertion to the body, enabling portable health monitoring and advanced disease diagnosis. Lastly, we summarize the most recent practical applications of advanced implantable devices for human health care, highlighting their potential for immediate commercialization and clinical uses.

## Highlights

- This article summarizes the recent advances in wireless, batteryless, implantable electronics for continuous physiological monitoring.
- The critical factors that affect the design of implantable electronics for biosensing are discussed.
- The recent progress of material research for developing various implantable devices is summarized.
- This article reviews various biomedical applications of implantable devices for human healthcare.

## Graphical abstract

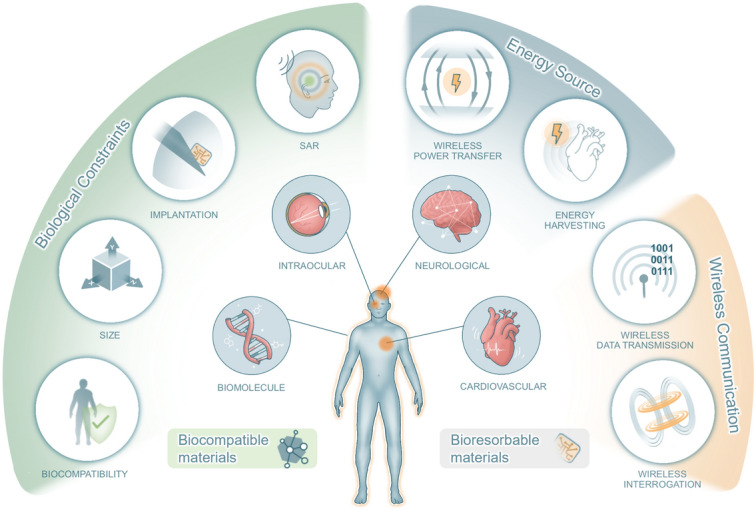

## Introduction

Electronic interfaces with living biological systems introduce the basis for various devices that monitor the behavior of different biological systems. Beyond understanding physiological behaviors, advances in bioelectronic interfaces have a broad range of applications, from medical diagnostics to drug discovery, physical activity monitoring, glucose level control, food safety, process control, and environmental monitoring, to defense and security applications [1]. Using bioelectronic interfaces to create biosensors allows translation of biochemical, bioelectrical, and biomechanical signals into standard electrical signals, which a wide range of electronic devices can interpret. Therefore, these devices are essential to understand, monitor, and analyze all biological phenomena. In particular, implantable biosensors are receiving great attention as devices that are capable of monitoring physical signals from inside the human body, showing promising results in monitoring neural activity [2, 3], cardiovascular signals [4–9] prosthetic integrity [10–12], organ behavior [13–15], and others. This is especially due to their ability to continuously monitor bioindicators, ease of use, high specificity, processing speed, low power requirements, and portability [16]. This review discusses highly biocompatible, battery-free, wireless, implantable sensor systems that enable the understanding, diagnosis, and treatment of in vivo phenomena.

As implantable devices are in direct contact with biological tissue and fluids, they face several biological constraints. These devices are in a harsh in vivo environment, being in direct contact with biological tissues and fluids. Moreover, they can restrict any bodily functions, cause an aggravated immune response, or damage the surrounding tissue. Therefore, it is of the uppermost importance to have materials that are biocompatible surrounding the device. Moreover, any mismatch in the mechanical properties between the device and the soft biological tissue can lead to complications. These may include inflammation, degradation, and tissue damage. Recent progress on soft materials shows its possibility of allowing for better conformity with tissue, reducing the inflammatory response created by the mechanic mismatch between implanted devices and biological tissues [17–19]. Moreover, using soft material can also reduce motion artifacts due to the mismatch of the mechanical modulus of implanted devices and the surrounding tissue [20]. Another class of materials gathering a lot of attention for fabricating implantable devices is bioresorbable materials, which degrade over time, leaving little to no traces behind [21]. This is highly relevant to devices that are only needed for short-term monitoring, as surgical removal procedures can be avoided, and infection risks can be mitigated.

Wired systems composed of electrical wiring, fluidic channel, or optical cable are commonly used in many medical systems as a simple, easy way to achieve the functional maturity of the device. However, the mechanical inconsistency with soft living tissue and the restricted operational modes could limit anatomical versatility, create functional constraints, and impair long-term applications. For example, pacemaker leads are known for being the most problematic component of the device [22], as they are prone to breaking, causing infections, venous obstructions, increasing the risk of thrombosis, and tissue perforation [23–26]. Pacemaker leads also cause issues with magnetic resonance imaging (MRI), as the presence of strong magnets will lead to impedance and signal changes, and heating of the lead tips, which could potentially cause damage to the surrounding tissue [27]. Leadless pacemakers have been shown to have a lower complication rate in the long term and allow for a simpler implant procedure with the use of catheters [22, 28]. Wire placement in dynamic regions presents another challenge, as the resulting tension and stress can induce wire breakages. Studies involving patients with deep brain stimulation implantable devices have reported instances of broken wires. These instances occurred when the implant's connector was located below the mastoid and never when positioned at the calvarium, as a location closer to the neck [29].

The use of wireless communication eliminates the need for a wired connection or device retrieval for data acquisition, minimizing physical contact and interference with living organisms, enabling less invasive and continuous monitoring. This unique feature enables data collection from direct contact with the target organ or tissue with continuous, long-term monitoring, regardless of the patient’s physiological state [30]. Thus, implantable devices offer more accurate and complete data than non-implantable devices and most imaging techniques, essential for diagnosing certain medical conditions [30, 31]. Moreover, with the increase of at-home monitoring solutions that can monitor the patient throughout their daily lives has created a need for a way to collect data from the patient’s body without any intervention. The usage of wireless data communication is the prime candidate to achieve this, as the risk of infection, the need to access the devices to reprogram them, and the need for the patient to perform any procedures in the implantable devices are reduced. Another advantage is the possibility of using standard communication protocols allowing for the connection with the patient’s smartphone, causing fewer changes to the patient’s daily lives compared to having them to wear a dedicated medical device. Additionally, in the case of animal trials, implantable devices are able to achieve a greater range of motion, and fewer interventions are needed to care for the animals, leading to more realistic results.

Batteries have been used since the inception of the first implantable devices and are still used today in most commercially available devices [32]. However, when dealing with complete and long-term implantation, the use of batteries in vivo can potentially cause serious health issues. Battery implantation requires a subsequent invasive surgical procedure for their replacement and will curb the device’s long-term usage, implant locations, and potential implant procedure because of its chemical hazards and physical constraints [32, 33]. Although advances in electronics over recent decades have resulted in substantial reductions in size, improved capacities, and power consumption, the same exponential growth does not hold for battery technology. The sluggish improvement in the energy density of batteries presents a notable limitation in the evolution of implantable devices [34]. The bulky size of these batteries hinders numerous applications that require smaller implants, such as those used in neurological and cardiovascular procedures, especially when long-term devices are needed, as increasing the battery’s capacity will usually significantly increase its dimensions. This limitation is a major roadblock in the current efforts to miniaturize implantable devices. Additionally, with the increasing use of implantable devices, the use of devices with short lifespans will severely impact their usage in the case of young patients, who will need to undergo multiple surgical procedures throughout their lives for implant replacement. Moreover, most commercially available batteries are rigid, which severely impacts the device’s capacity to conform to the soft biological tissue. This limitation also causes issues when designing devices that can fold or be compacted during the implantation procedure to be later expanded, as the battery cell cannot be expanded in the same way. Additionally, the implantation procedure needs to be designed to not interfere with the battery, as any perforations or additional stress can lead to its hazardous chemical content leaking or reactions, leading to serious health complications for the patient.

Therefore, the creation of a batteryless, implantable system is a fundamental approach to achieving a fully wireless and lightweight system without impairing the patient’s daily life, as it can reduce the system size and eliminate the need for subsequent surgery to perform battery replacement or device removal [35, 36]. To achieve a batteryless system, the most straightforward approach is the use of passive devices, as they do not have any power requirements. But the use of active devices, enabling more complex functionalities, is also possible with the help of energy harvesting, harvesting energy available inside [37–42] or outside [43–46] the body, or wireless power transfer (WPT). Careful consideration of each technique’s benefits and drawbacks is needed for choosing the ideal strategy for powering the device.

In addition, it is necessary to select an effective wireless communication strategy for the reliable transmission of data to external devices, as different techniques will be better suited for different implantation depths, data rates, and precision. Data retrieval can be achieved by either interrogating the properties of the device or by having it transmit the data to the user. A device can be designed to change its shape or size in response to the biosignal of interest, enabling it to be imaged by ultrasound, allowing an ultrasound system to image it [47–49]. Another property of devices that can be changed is their resonance frequency, by connecting a capacitive sensor to an inductor, which can be interrogated by a coupled antenna from outside the body [50–53]. As for having the device to actively transmit the data, one could use NFC, Bluetooth, RFID, ISM bands, backscattering, infrared, and ultrasound transducers, each with its own advantages and drawbacks. The attenuation of the signal by biological tissue also needs to be considered, as some commonly used frequencies, such as 2.4 GHz, will present issues transmitting data from implants deep in the body. Furthermore, the absorption of energy by biological tissues will also need to be considered, as this can lead to thermal damage to them, limiting the power of the signal transmitted. Despite there are multiple ways to achieve wireless data communication, optimal design choices are fundamental to enable reliable and continuous data collection without intervention.

**Fig. 1:**
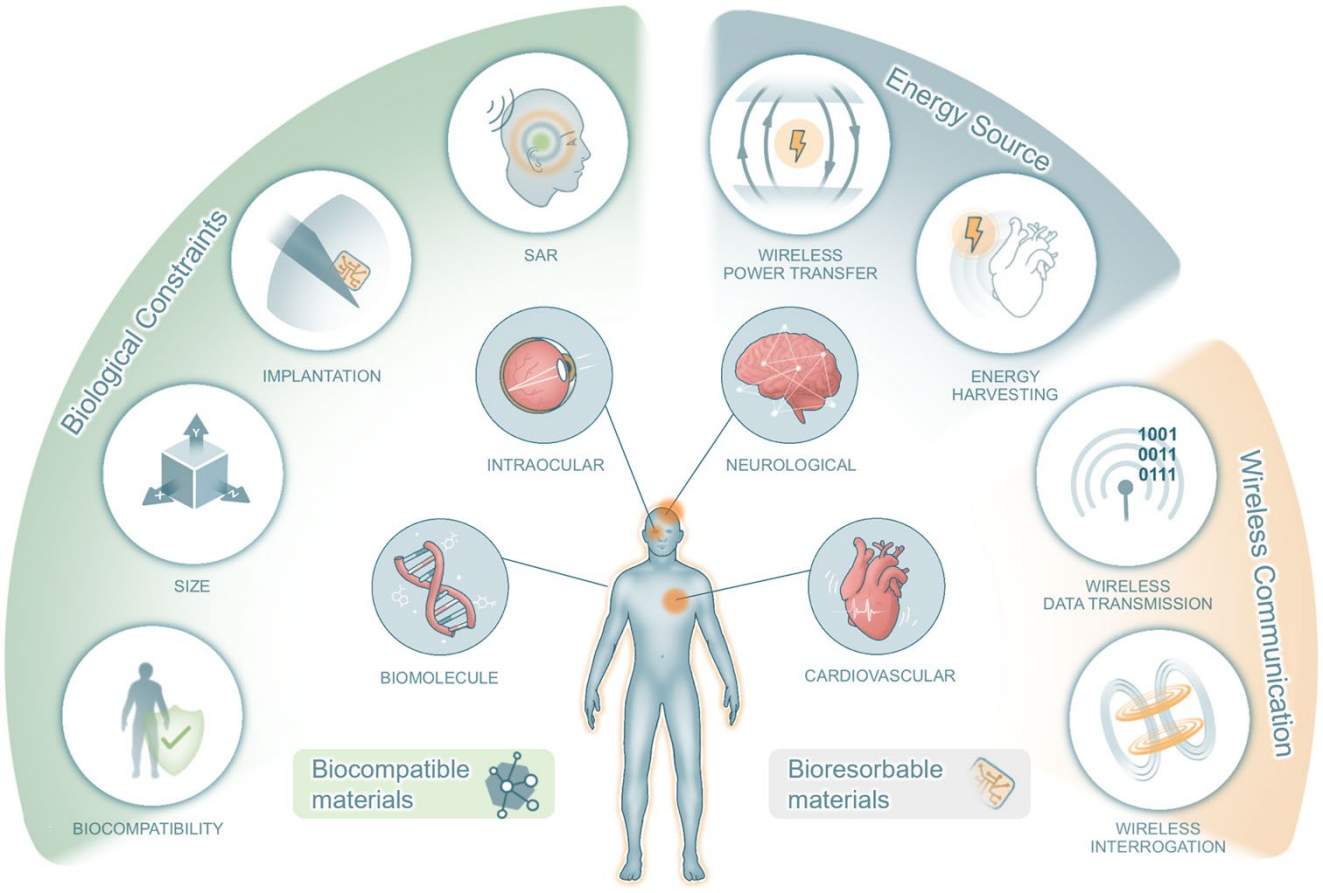
Schematic illustration of the design considerations and applications of wireless, batteryless, implantable electronics

Here, this article reviews the recent advances in batteryless, fully wireless, implantable devices and discusses their challenges (Fig. 1). In terms of biocompatibility and functionality, this work discusses materials selection and engineering approaches regarding the bioelectronic interface. This study will also review strategies utilized for implant size reduction, to achieve less invasive implantation, and to deal with SAR limitations. Key engineering approaches toward fully wireless communication will also be included, discussing how to interrogate implantable devices or have them transmit data. Moreover, strategies on how to power devices without using batteries will also be discussed, focusing on wireless power transfer and energy harvesting. Various practical applications of implantable devices are reviewed, highlighting engineering strategies and device performances of recent cardiovascular, neurological, intraocular, biomolecular sensing studies.

## Design Considerations

### Biological Constraints

Implantable devices are subjected to stricter biological constraints than other biomedical devices since the implanted biosensors will be in direct contact with different biological tissues. Therefore, they need to be biocompatible, both biologically and physically. Each different implantation location offers unique dimensional limitations, usually requiring careful miniaturization of the device. Moreover, each location has its own challenges for the insertion of the device, especially with the preference for minimally invasive procedures. Furthermore, the electrical limitations of the surrounding tissue also need to be respected by paying close attention to the SAR limits.

#### Biocompatibility

**Fig. 2:**
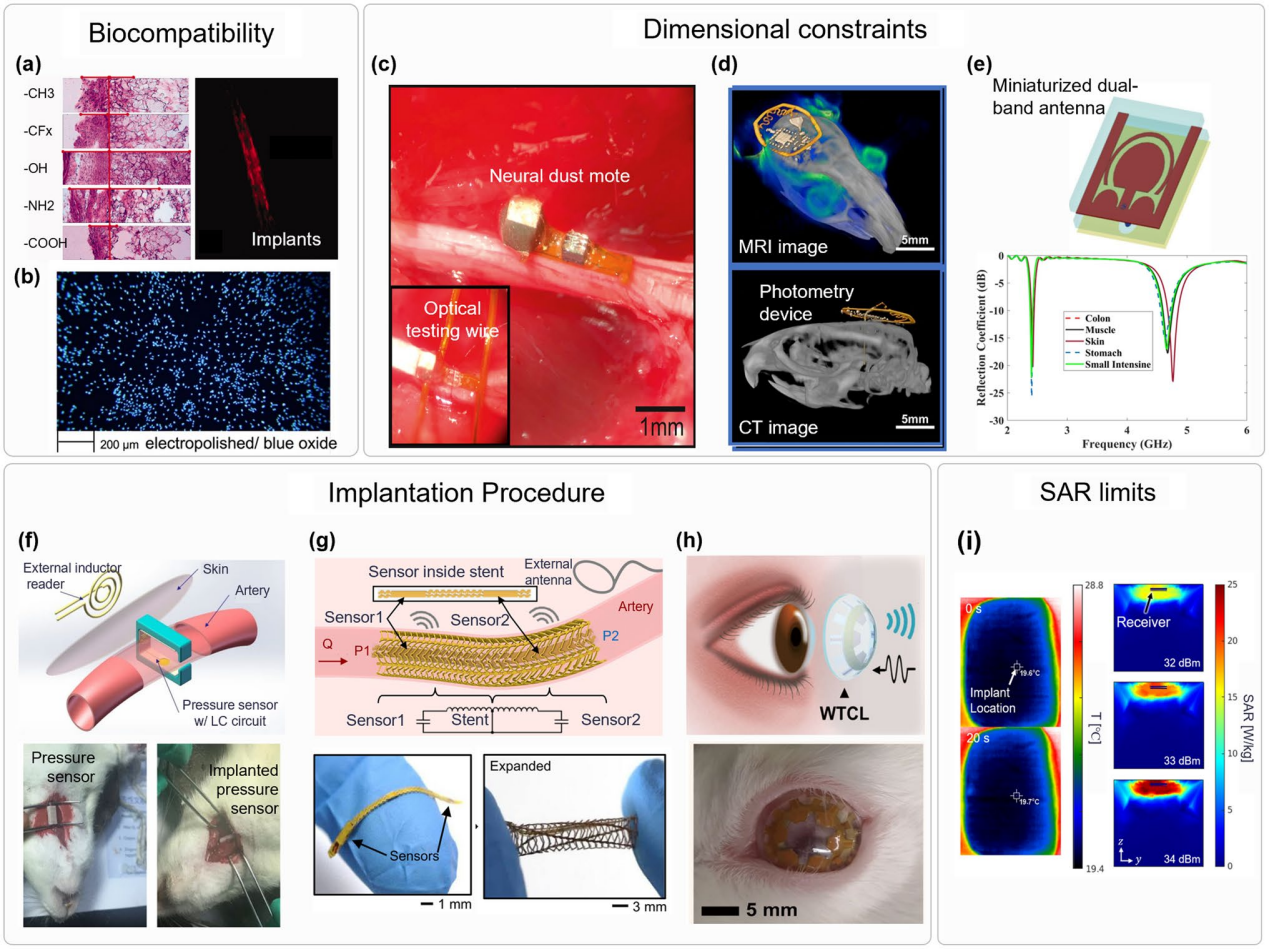
Design consideration for the implantable electronics. Biological constraints: biocompatibility, dimensional constraints, implantation procedure, and SAR limits. **a** Tissue response and immunohistochemical analysis for implants with different functional groups. Reproduced with permission [57]. Copyright 2007, Wiley-VCH. **b** Image of DAPI-stained human cells cultivated on electropolished/blue oxide nitinol. Reproduced with creative commons license [58]. **c** Neural dust module implanted in a rat nerve. Reproduced with permission [2]. Copyright 2016, Elsevier. **d** Combined MRI and CT scan of a mouse after implantation of a small photometry device. Reproduced with creative commons license [3]. **e** Structure and S11 parameter of a miniaturized dual-band antenna. Reproduced with permission [59]. Copyright 2022, Elsevier. **f** Extra-arterial implantable device and its implantation procedure. Reproduced with permission [5]. Copyright 2020, Springer Nature. **g** Implantable pressure sensors designed to fit into an inductive stent. Reproduced with creative commons license [4]. **h** Sensor for monitoring IOP integrated into a contact lens. Reproduced with creative commons license [60]. **i** Safety test in a piece of pork and SAR simulation of implantable device. Reproduced with creative commons license [61]

It is of the uppermost importance for implantable devices to be safe to use and not to entice any kind of unwanted response, such as inflammation, irritation, foreign body rejection, or immune responses. Such responses can vary on level from minor immunological response with accumulation of defensive cells around the implant to impairing the correct functioning of nearby organs [54]. Thus, selecting materials that will provide a safe interface between the device and the biological tissue is one of the most important design steps for implantable devices. The level at which the material is effective at being implanted in vivo without the occurrence of major harmful local or systemic responses is called the biocompatibility [55]. To reduce interactions and improve the implant's biocompatibility, the engineering of the implant's chemical properties, especially surface chemical properties, plays a key role [56]. To minimize protein interaction, researchers have been placing efforts into developing implantation surfaces with uniform functional groups. Surfaces with a carboxyl functional group, for example, have been shown to attenuate inflammatory responses and reduce fibrotic capsule formation in vivo, while surfaces with an amine functional group trigger acute inflammatory responses and fibrotic capsule formation, as illustrated in Fig. 2a [57]. Ion release and corrosion must also be considered when selecting the best implant material to increase biocompatibility [58]. For example, the use of electropolishing of nitinol, as shown in Fig. 2b, provides a more homogenous surface and protects against corrosion and ion release.

#### Dimensional Constraints

Implantable sensors have been used in multiple locations subjected to different size constraints, such as subdermal [44] or neural implants [2]. But not only the location of the implant matters, as implants being targeted to animals, such as rats, will suffer from even further reduction of the available space. Despite some applications having tighter dimensional constraints than others, a lot of effort has been applied to miniaturizing implants, even in areas that do not necessarily need smaller implants. This trend can be explained by smaller implants being usually less noticeable, easier to implant, and less aggressive to the body due to the reduced interface between tissue and implant, which reduces biocompatibility issues. With reduced sizes, the sensors can also be delivered using less invasive methods, such as catheterization or with the use of a needle. A great example of an area that greatly benefits from smaller implants is neurology. The brain and the skull do not allow for big implants to be installed, especially as the brain tissue is fragile, so implants should present good conformity with it. Moreover, considering the number of signals that are present in the brain, smaller sensors will allow for collection of even more channels of data, helping to further neurological research. A great example of successful miniaturization of a neurological sensor is the work conducted by Seo et al. in which the group created a device named neural dust, shown in Fig. 2c, to collect electromyogram (EMG) and electroneurogram (ENG) signals [2]. With the use of ultrasound to power the device and transmit data, they were able to achieve an implant that measures only 0.8 × 3 × 1 mm, allowing for data collection on small-diameter nerves. Figure 2d shows a device that is implanted on the brain of rats [3]. This device was designed to conformably fit the rat’s brain and to be able to transmit data using IR. Electronic components with a small footprint were chosen in a way to minimize the device’s area, consuming only 0.21 cm^2^. Ocular implants are another area that benefits from miniaturization, as the eyes require implants with a small surface area to not obstruct vision and that is also thin to not interfere with eye lubrification. Intraocular implants to measure the pressure inside the eye to track glaucoma progression recently were able to reach a sub-mm^3^ volume. Bhamra et al. developed an implantable intraocular pressure monitor microsystem (IMM) consisting of a powering coil, an antenna, a MEMS pressure sensor, and a pressure sensing IC that only occupies 0.38 mm^3^, being able to measure glaucoma progression in rodents [61]. This small volume was achieved thanks to the use of a piezoresistive pressure sensor, which is 14 times smaller than capacitive ones. The use of wireless energy transfer and wireless communication is fundamental to be able to achieve miniaturized implants. Lee et al. [62] have shown that removing the batteries, data storage, and sampling from the system is able to greatly reduce the system size. The group obtained a sensor that only occupies 3 × 6 mm^2^ with the use of fluorescent hydrogels, with a reading distance of up to 2 mm. Reduction in size of the system is especially difficult; it works in low frequencies of the electromagnetic spectrum, below a few GHz. The optimum performance of antennas is usually achieved at half the wavelength, which for 10 GHz is around 1.5 cm. But most biomedical systems operate at a lower frequency than this, with the majority of them working with frequencies lower than 10 MHz, at which point the electromagnetic waves can better penetrate the body, with low tissue absorption, and more recently with frequencies in the UHF band (300 MHz–3 GHz), with high power transfer efficiency [59]. Therefore, research in reducing the size of coils and antennas is essential for biomedical implants. In the case of RF systems, multiple efforts have been made to miniaturize the receiver antenna. A paper [63] showed a novel design for a dual-band antenna is proposed, receiving signals at 2.4 GHz and reflecting signals at 4.8 GHz. This antenna, shown in Fig. 2e, has a volume of 8.5 × 8.5 × 1.27 mm^3^, presenting a novel U-shaped part and three semicircles that simplify manufacturing and reduce size. In Ref. [60], an antenna that can operate at 2% wavelength, with a size of $\uppi \times (3\times 1.5)$×1 mm^3^, is demonstrated. In this work, Ma et al. proposed a coupled-ring antenna with a focus on brain implants, providing a reading range of 60 cm when the implant is placed 16 mm deep.

The dimensional constraints imposed by the different organs not only affect the size of the possible implants but also impose restrictions on their shape and mechanical properties. The implant's surface and structure must be designed to conform to the tissue walls. The implant should have an external shape and properties that should be compatible with the target tissue to avoid trauma [54]. Moreover, its structure should allow for the following changes in the tissue shape; thus, the mechanical properties of the implant should match the ones from the tissue. For example, in the case of blood vessels, the implant should avoid interfering with their ability to compress or dilate blood flow [4].

The use of soft and flexible materials and designs allows for an improved mechanical consistency between the implant and the soft biological tissue. The advancements in this type of materials will allow for the development of implantable devices with little to no impact on the organism, as a good mechanical interface between the implantable device and soft tissue can be obtained [64]. Moreover, flexible devices can also be obtained through the use of designs that can stretch, such as buckling, serpentines, origami, and textile designs [65]. In [9], Herbert et al. developed a soft strain sensor to detect early signs of restenosis. The device was made to be highly conformal with arteries through the use of elastomer encapsulation and the use of a serpentine design to allow non-soft materials to be stretched.

#### Implantation Procedure

To design clinically applicable implantable devices, one needs to take into consideration how it is going to be implanted. The risks for patients who undergo complex surgical procedures to insert a biosensor will probably outweigh the benefits provided by the diagnostic information provided by the devices. Therefore, is detrimental that researchers give priority to minimally invasive procedures and consider the benefits provided by the device when considering more invasive techniques. For example, cardiovascular sensors that are implanted into arteries can be placed around the artery or inside of it. Extra-arterial sensors can be less prone to blood coagulation and infection [5], but they require direct access to the external walls of the artery, while internal sensors can be delivered with the use of catheters. Hu et al. [5] developed an arterial cuff with a pressure sensor that can be read wirelessly and conducted in vivo experiments on rats, but surgery to expose the rat’s carotid artery was needed, as illustrated in Fig. 2f. Different catheter deployed arterial sensing systems were developed [4–9]. Those have additional requirements, as they not only need to be compatible with the inside of the artery, but also need to be deployed with the expansion of the catheter’s balloon, usually coupled with a stent. The stent can also be used as a part of the system. Islam et al. [6] developed a stent that can harvest ultrasound energy by using PVDF. The smart stent can generate energy to power the data collection and transmission system. A geometrical pattern was engraved into the surface of the stent to increase the vibration and obtain a negative Poisson’s ratio, allowing for expansion. An inductive stent, shown in Fig. 2g, was also developed and connected to a pressure capacitive sensor, forming an LC system, to collect data wirelessly [4, 7]. The use of a pattern that forms multiple turns of a coil throughout the stent’s body was used to make it behave as an inductor. The coils are initially folded to allow for expansion, enabling the use of a catheter, and PI links are used to keep structural integrity. Like in the case of stents, the addition of a diagnostic feature to a therapeutic implantable device is also a good way to avoid unnecessary surgeries. Sensors can also be used to check the structural integrity of the therapeutic device, or to check whether any post-surgery complications occur. In Ref. [10], a fractal RFID sensor is used to detect cracks in metal prostheses, being able to detect cracks as small as 600 um. In the case of prosthetic joints, loosening can also be wirelessly sensed using inductive sensors with an LC topology [11]. Other devices have focused on measuring the joint’s contact forces and moments to assist researchers and manufacturers in future prosthetic design [12]. In the case of intraocular pressure monitoring, Yang et al. presented a capacitive pressure sensor that is integrated into the curved surface of a contact lens, shown in Fig. 2h [66]. Even sutures have been transformed into wireless sensing devices. By using two stretchable fibers to form a capacitive strain sensor that can be connected to an inductor, Lee et al. [67] demonstrated the detection of strain in ligaments wirelessly.

#### SAR Limits

Most wireless devices need to be able to transfer their data and receive power from outside the body. To reach the implant, the signals need to travel through tissue, which will cause them to be attenuated because the tissue will absorb part of it. The specific absorption rate (SAR) is the amount of energy absorbed from electromagnetic waves by the body. This quantity is dependent on the both tissue and the frequency of the signal [68]. Wireless devices need to consider the SAR safety limits according to their implant location to avoid generating excessive heat and damaging the surrounding tissue. Moreover, some organs, such as the eye or testis, are more likely to be damaged by heat, while others have more resilience. SAR safety limitations will be one of the biggest constraints for the implantation depth of the device, as there will be more tissue absorbing energy, increasing SAR and reducing the energy delivered to the implantable sensor. Devices such as the one presented in Ref. [62] were optimized to deliver the most power possible to the sensor while respecting the SAR limits. SAR calculations and measurements were included in Ref. [62] and can be followed by future studies that aim to certify their devices operate within the safety limits. Simulations and tests of the device’s SAR are shown in Fig. 2i. An antenna array was developed in Ref. [69] to simultaneously communicate with up to eight devices using an RFID-based link. The use of an array was a way to optimize the energy transferred to each device while still observing the SAR limits.

### Energy Source

Wireless power transfer in implantable devices refers to transferring energy from an external source to the implant without physical connectors or wires. An alternative power source needs to be included to avoid using batteries to power the device. To achieve this, an external transmitter can be used to transfer power to the implantable device. This allows for more control of the power provided to the device and has higher power capabilities. Another way to provide power to implantable devices is to use energy harvesters to transform energy contained in the body or gathered from the environment. This mechanism enables the devices to function without a transmitter, thus facilitating more continuous operation; however, it also tends to be less reliable. Table 1 compares the implantable medical devices discussed in this section according to their power, size, principle, and implantation location. Each principle has its advantages and disadvantages, which will be discussed in depth in their own sections. In comparison, the power transmission techniques (inductive coupling, ultrasound, capacitive coupling) usually offer a higher power budget compared to the energy harvesting techniques (piezoelectric, triboelectric nanogenerators, electromagnetic induction, photovoltaic generators, thermoelectric, pyroelectric, biochemical), but these have the need of an external device to send power to the implantable device, while the later are able to harvest energy from the body or from the environment. Capacitive coupling is the method that allows for a more significant amount of power to be used by the device, but is limited to low-depth subdermal implants. This is followed by inductive coupling, which can offer a good power budget for medium-depth implants. In comparison, ultrasound power transfer is better at powering devices that have deeper implantation or are smaller and are also not limited by SAR. In the case of devices with power harvesters, photovoltaic harvesters seem to offer the greatest amount of power, but are limited to lower implantation depths and only generate power when the environment has a light source. In comparison, energy harvesters that can collect energy from motion are able to be implanted deeper and collect energy throughout the entire day. Electromagnetic induction seems to be the type of implant that causes more limitations to the organism, and piezoelectric generators require the most space. Still, they usually offer more power than triboelectric nanogenerators. Thermoelectric and pyroelectric generators have been shown to be able to also offer a good power budget when implanted sub dermally, but few implantable devices were designed using these methods. Biochemical generators have also been shown to offer a high amount of power compared to other energy harvesters and can potentially be implanted deep into the body.

**Table 1:** Comparison of different powering principles for implantable devices

Principle	Implant location	Improvement	Range (mm)	Size (mm)	Power	Efficiency (%)	References
Inductive coupling	Heart	Increase in Q factor	250	45 × 2.5 × 1.3	2.5 mW	–	[70]
	Eye	Adjustment to coil misalignment	–	0.38 mm^3^	48.9 $\upmu$W	4.89	[61]
	–	Constant output voltage	50	900 mm^2^	–	76.9	[71]
	Subdermal	Size reduction due to resonance	90	10.5 × 7	17.64 mW	0.6 (worst case)	[3]
	Subdermal	Size reduction	5	6 × 11 × 1.7	40.9 $\upmu$W	4.1	[72]
	–	Split ring resonators	22	45 × 45	–	87.9	[73]
Ultrasound	Brain	Materials	–	13.5 × 9.6 × 2.1	280 $\upmu$W	–	[74]
	Artery	Pattern in PVDF	–	30 × 10 × 10	230 $\upmu$W	11.5	[6]
	–	Triboelectric ultrasound harvester	10	40 × 40 × 1	98.6 $\upmu$W	–	[75]
	–	TENG with attached electrode	–	60 × 40	> 1 mW	–	[76]
Capacitive coupling	Subdermal	Operating frequency optimization	3	20 × 20	100 mW	56	[77]
	Subdermal	Reduction in SAR	5	23 × 40	105.5 mW	28.4	[78]
	Subdermal	Data transfer integration	3	20 × 20	12 mW	36	[79]
	Subdermal	Auto-resonance tuning	2	20 × 20	150 mW	54	[80]
Piezoelectric harvester	Aorta	Feasibility	–	56 × 56 × 0.2	681 nW	–	[37]
	Heart	Kirigami	–	28 × 60	228 nW	–	[38]
	Heart	Kirigami	–	40 × 30	2.4 $\upmu$W	–	[39]
TENG harvester	Abdomen	Stretchable	–	11 × 30	120 nW	–	[40]
	–	Ion implantation	–	20 × 20	70 $\upmu$W	–	[41]
Electromagnetic induction	Heart	Feasibility	–	21 × 5 × 5	1.7 $\upmu$W	–	[42]
PV harvester	Subcutaneous	Infrared	–	1 × 1.2	134 $\upmu$ W mm^−2^	12.24	[45]
	Subcutaneous	Use of external device	–	11.1 mm^2^	74.1 $\upmu$W mm^−2^	–	[46]
Thermoelectric	Subcutaneous	Feasibility	–	15 × 15 × 3.9	14.4 $\upmu$W	–	[81]
Pyroelectric	Subcutaneous	Feasibility	–	20 × 20	–	–	[82]
Biochemical	Ex vivo	Feasibility	–	3.01 × 3.01	0.74 $\upmu$W	–	[83]
	Abdomen	Integration	–	–	0.08 mW	–	[84]

#### Wireless Power Transfer

**Fig. 3:**
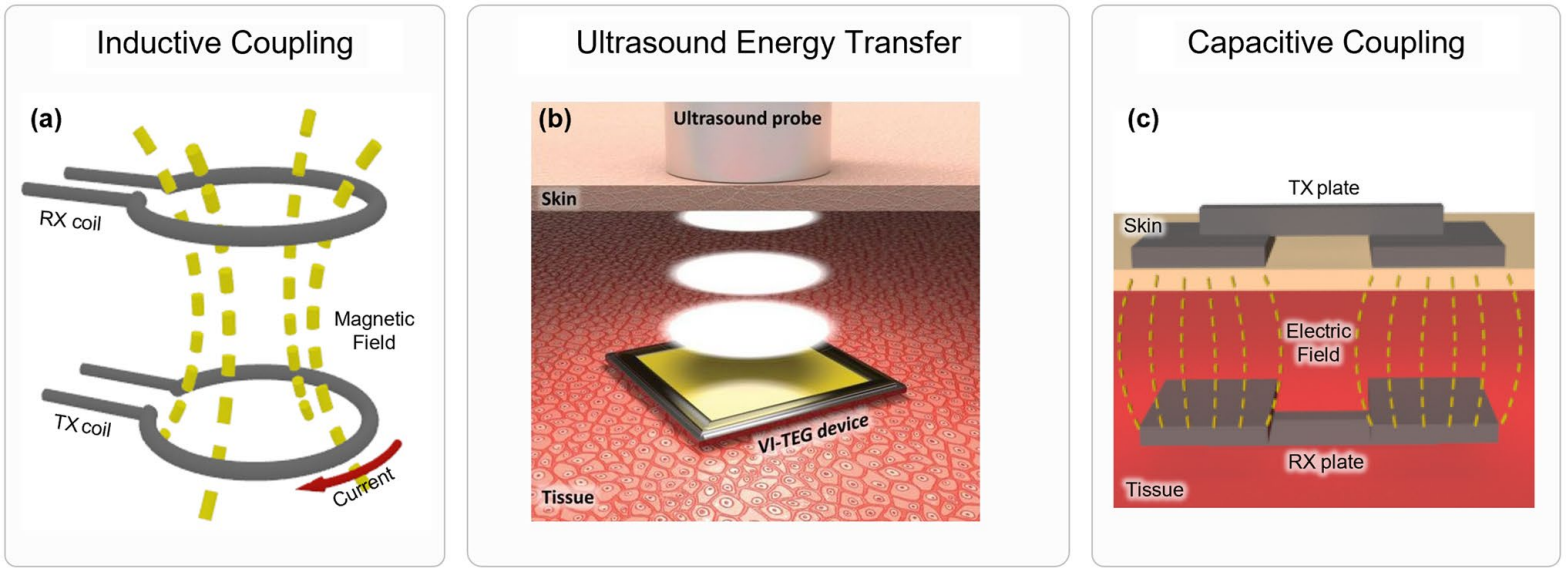
Working principle of wireless power transfer techniques. **a** Schematic of inductive coupling in which a current in a coil, represented by the red arrow, creates a magnetic field going through a secondary coil. **b** Schematic of utilization of an ultrasound probe to send energy to implanted device. Reproduced with permission [72]. Copyright 2019, AAAS. **c** Schematic of capacitive coupling, in which internal and external electrodes forms capacitors using biological tissue as the dielectric

The wireless power transfer (WPT) technique has been used in numerous fields to avoid the use of wires or to minimize and cheapen commercially available devices by avoiding the use of batteries. This method has been applied to RFID tags for product tracking, satellite communication, cellphone charging, and many others. This has also been extensively applied in the case of medical devices, as the use of batteries is one of the main constraints for dimensional reduction and long-term applications of implantable devices. WPT is defined as the transfer of electrical energy without any wires, and in its majority, consists of the use of time-varying electromagnetic fields created by an external source to induct currents in the implantable devices themselves. There are multiple methods of WPT that have been used to power medical devices, as shown in Fig. 3. Those can be classified into a few subcategories: inductive coupling, capacitive coupling, and ultrasound. Inductive coupling is the most widely used method of WPT, being the most established and studied method of transferring energy. This method concentrates most implantable device publications that utilize wireless power transfer (WPT).

##### Inductive Coupling

Inductive coupling is based on the power transfer from one coil to another. One of the coils will have an oscillating current applied to it, creating a varying magnetic field in the second coil, generating an electromagnetic field (EMF) on it that can be used to power the medical device. The coil with the changing current is commonly called the transmitting coil (TX), and the coil that has an EMF induced is commonly referred as the receiving coil (RX). A simplified diagram of this process is shown Fig. 3a. The power transfer capability of this technique will rely on several factors. Firstly, it will depend on the transmitter power, which is restricted by SAR. Additionally, it will be influenced by the separation between the coils, which is constrained by implant locations. The frequency used in the TX coil will also play a role, as it will be limited by reflection and absorption in the tissue. Lastly, the coupling factor, determined by the distance, alignment, and design of the TX and RX coils, will also have an impact. John et al. [70] propose different techniques to improve the efficiency of inductive coupling, such as the use of a ferrite core, and the correct selection of radius and thickness of the coil. Del Bono et al. [85] proposed a system to limit biological tissue heating by using a closed loop to limit or increase the power delivered when a different efficiency is obtained. Another challenge with inductive coupling in implantable devices is dealing with the implantation depth. Some implants need to be implanted deep within the body, limiting the efficiency of the wireless link and increasing SAR. To deal with this, Alghrairi et al. proposed a system using three coils to transmit power to an implantable stent [86]. The group was able to achieve an efficiency of 82% in 20 mm of air. One of the main issues with inductive coupling is coil misalignment, as the implants can change place or shift, and there is no feedback when adjusting the alignment of the external coil. Another issue is when the devices are used in free-roaming animal studies, as the alignment of the coils is going to be constantly changing. To overcome this, Bhamra et al. created a matching network that dynamically adjusts according to a drop in efficiency due to coil misalignment [61]. By tracking changes in the voltage supplied to the circuit during different clock cycle, the implant can adjust its capacitor network to increase the efficiency of the power transfer despite the mismatch in orientation of the coils. The optimization of the coupling coefficient with coil misalignment was conducted in [87]. Bao et al. found that using a planar spiral coil as the transmitting coil and a solenoid coil as the receiving one achieved a power transfer efficiency of 44.14% and a mean power delivered of 145.38 mW. As another way to deal with coil misalignment, Basir et al. proposed a sphere-shaped coil that greatly improved the system’s performance under more extreme misalignment conditions [88]. Another way to achieve higher efficiency with inductive coupling is using resonant inductive coupling, often also called magnetic resonance coupling. In this case, the resonance effect on the receiver side is used to reduce the resistive losses in the coil, which get higher with the distance between TX and RX. To reduce the coil impedance, a capacitor is placed in parallel to the coil making them resonate at the frequency used to excite the TX coil. The coil can also be optimized to self-resonate at a certain frequency by controlling its self-capacitance. Lin et al. [71] propose multiple resonant topologies that can achieve output voltages that are independent of the coupling coefficient. This method offers benefits for implants that face tighter SAR limitations, as it will require less energy due to its higher efficiency. Burton et al. developed a subdermal implant to record brain signals that use a small coil, dramatically reducing the implant size with the use of resonance [3]. Biswas et al. [72] developed an optogenetic device that can be powered by a small 6 × 6 mm^2^ square inductor and a resonance capacitor. To transform the AC power received by the coil to DC power to light the mini-LED, a half-wave rectifier composed by a Schottky diode, and a capacitor was used. To achieve strong coupling, Kurs et al. have proposed to use self-resonant coils coupled to the TX and the RX coils to drastically increase efficiency [89]. This research has provoked interest in the magnetic resonance field. Their design can offer profound impacts in the wireless-powered implantable device field, as it can extend the operating distance of most devices. Wang et al. proposed a self-resonator split-ring loop that was able to achieve 87.9% WPT efficiency at a 22 mm distance [73]. Their antenna design can be easily manufactured as a planar coil and be easily incorporated into other devices. Hua et al. conducted in vivo tests using magnetic resonance-based WPT with a sensor implanted inside a pig, validating the use of this technology for implants [90].

##### Ultrasound Energy Transfer

The use of acoustic waves to transmit energy was initially proposed by Cochran et al. in 1985 [37]. Since then, multiple devices have been created to explore this energy source. The use of ultrasound allows for much lower tissue absorption when compared to electromagnetic radiation. Moreover, it performs better than inductive coupling when dealing with smaller implants or higher reading distances [91]. Because of the significant difference between the speed of light and sound, acoustic waves have much smaller wavelengths of the same frequency, allowing for the design of much smaller transmitters and receivers. Compared to inductive coupling, ultrasound energy transfer is usually used for low-power implants. This technology can also be used as an energy harvester, but most of the focus in this field has been on the use of a transmitter outside the body, as it can offer more and more reliable power to the device [92]. This process is illustrated in Fig. 3b. Most devices use the piezoelectric capabilities of materials to capture energy from ultrasound. The piezoelectric effect is the energy generation in some solid materials due to mechanical stresses due to changes in its dipole moment. This can be obtained through the implantation of ions or the presence of asymmetrical charges on the material's crystal lattice. As sound is a mechanical wave, its propagation will move the particles inside the material, creating mechanical stresses and because of the piezoelectric effect, generating energy. Because ultrasound has much lower absorption in tissues than electromagnetic signals, it offers great potential for devices that are going to be implanted into tissues that have lower SAR limits. Therefore, it offers a great opportunity for devices to be implanted deep into the brain. Zhang et al. [74] designed a deep brain stimulation device that uses the piezoelectric electric effect to collect ultrasound energy with high power density. This device uses an SM-doped Pb(Mg_1/3_Nb_2/3_)O_3_-PbTiO_3_ crystal that can obtain up to 1.1 W cm^−2^ in vitro, which is much higher than the previous maximum, which was 60 mW cm^−2^. In Ref. [93], a flexible piezoelectric device was proposed that uses a multilayered design to improve its efficiency, achieving a maximum power of 13.13 mW underwater. Another advantage of ultrasound systems is that they are not dependent on metals like inductive coupling, allowing for the use of other material classes. This can allow for implantable devices that are traditionally dependent on metals to explore more flexible materials. Islam et al. [6] developed a stent that also collects ultrasound energy by replacing the metal that most stents are built by PVDF. Moreover, they also show that the usage of custom geometric patterns can allow for higher energy collection, due to increased surface vibrations. The main issue with piezoelectric devices that are going to be implanted in the body is their toxicity. Most piezoelectric materials contain lead, a material known for causing poisoning and polluting the environment. Polymers such as PVDF one, utilized in Ref. [6], or KNN as used in Refs. [94, 95], and other lead-free piezoelectric ceramics shown in Refs. [96–99] have been proposed as alternatives to avoid the toxicity problems. AlN-based piezoelectric devices have also been proposed as an alternative [100]. Other ways to collect energy from acoustic waves have also been proposed. Hinchet et al. [75] used ultrasound to displace a triboelectric generator’s membrane, generating power. This generator consists of a Cu/Au electrode as the primary electrode, a Cu electrode as the reference, and a membrane. When acoustic waves go through the device, the membrane is displaced touching the electrode. When this happens, the membrane becomes negatively charged, and when it moves again, the potential in the system is changed, creating a current. Another triboelectric ultrasound device has also been proposed, offering a 66% improvement with an attached electrode structure and being integrated into a flexible PCB instead of a rigid one. Recently, more interest has been gathered in the use of triboelectric nanogenerators for power transmission through ultrasound waves. An injectable and biodegradable device is shown in Ref. [101]. This device shows a highly miniaturized design, with dimensions of 2 × 0.2 × 0.05 cm^3^, and was able to provide 356.8 mV when implanted in vivo into a rat model. Lee et al. [102] showed an increase of 58.5% in the output voltage of the device with the of a porous structure of poly(3-hidroxybutyrate-*co*-3-hydroxyvalerate). The device was also biodegradable and was tested ex vivo in porcine tissue.

##### Capacitive Coupling

Capacitive power transfer is in its early stages of research being applied to medical devices, but it offers some advantages when compared with inductive coupling. Due to the electric fields being confined between the capacitive plates, it offers lower electromagnetic interference than inductive coupling [99]. Also, as it is capacitive based, it offers higher efficiency at higher frequencies due to its lower impedance. Moreover, its SAR values are lower than the inductive coupling [103]. Despite those advantages, capacitive coupling is only suited for implants that are near the skin due to dielectric losses. Moreover, it also has a lower efficiency than inductive coupling. The concept behind capacitive coupling consists of forming a capacitor by implanting a metal plate below the biological tissue to receive the power (RX), and another metal plate is placed on top of the body to transfer energy (TX), forming an electric field between them, with the biological tissue acting as the dielectric layer. When high-frequency signals are placed across a capacitor, its impedance is low, transferring most of the power to the other side. So, when using high-frequency signals, energy can be conducted through the skin. If two capacitors are used, one in the positive terminal of the device and one in the negative terminal, a complete circuit can be obtained, as shown in the diagram in Fig. 3c. There has been a research focus on making capacitive coupling implantable devices that are flexible, as those can better conform to the skin, offering better performance. In Ref. [77], they used copper patches encapsulated by PDMS to fabricate flexible electrodes. Moreover, the group also showed the feasibility and safety of delivering hundreds of milliwatts by optimizing the device's operating frequency of the device and succeed in attaining a power transfer efficiency (PTE) exceeding 50% in primate cadavers.

Researchers have been trying to improve the capabilities of capacitive coupling. To reduce SAR in capacitive coupling even further, Ref. [78] showed a capacitive coupling system operating at a low frequency (211 kHz) that could offer up to 290 mW with a PTE greater than 31%. Other researchers have also successfully used capacitive coupling to transmit both power and data. Koruprolu et al. [79] showed power delivery with an efficiency of up to 36% in biological tissue while using a hybrid of amplitude–frequency shift keying to transmit data simultaneously. Nag et al. [80] used capacitive coupling with flexible electrodes to transfer data and power simultaneously with a PTE that can reach up to 54% using automatic calibration and tuning of the carrier frequency.

#### Energy Harvesting

Implantable medical electronics can be powered by energy harvesters, which harvest energy from sources inside the body, such as respiration, heartbeat, or external sources. The main intention of energy harvesters is to collect power available in the device's environment, be it from the body or sources such as sound or light. Harvesting energy from motion can be obtained using triboelectric and piezoelectric nanogenerators or electromagnetic generators. Energy from outside the body can also be obtained using the transcutaneous approach, as optical and acoustic energy can be transferred through the skin. As those methods do not use RF or microwave, SAR limits do not apply to them, offering an advantage when compared to WPT. Moreover, it also allows for devices to be powered without an external source. This also causes energy harvesting to be less predictable and more variable. Energy harvesters also usually offer less power than WPT devices [104].

##### Kinematic Energy Harvesting

**Fig. 4:**
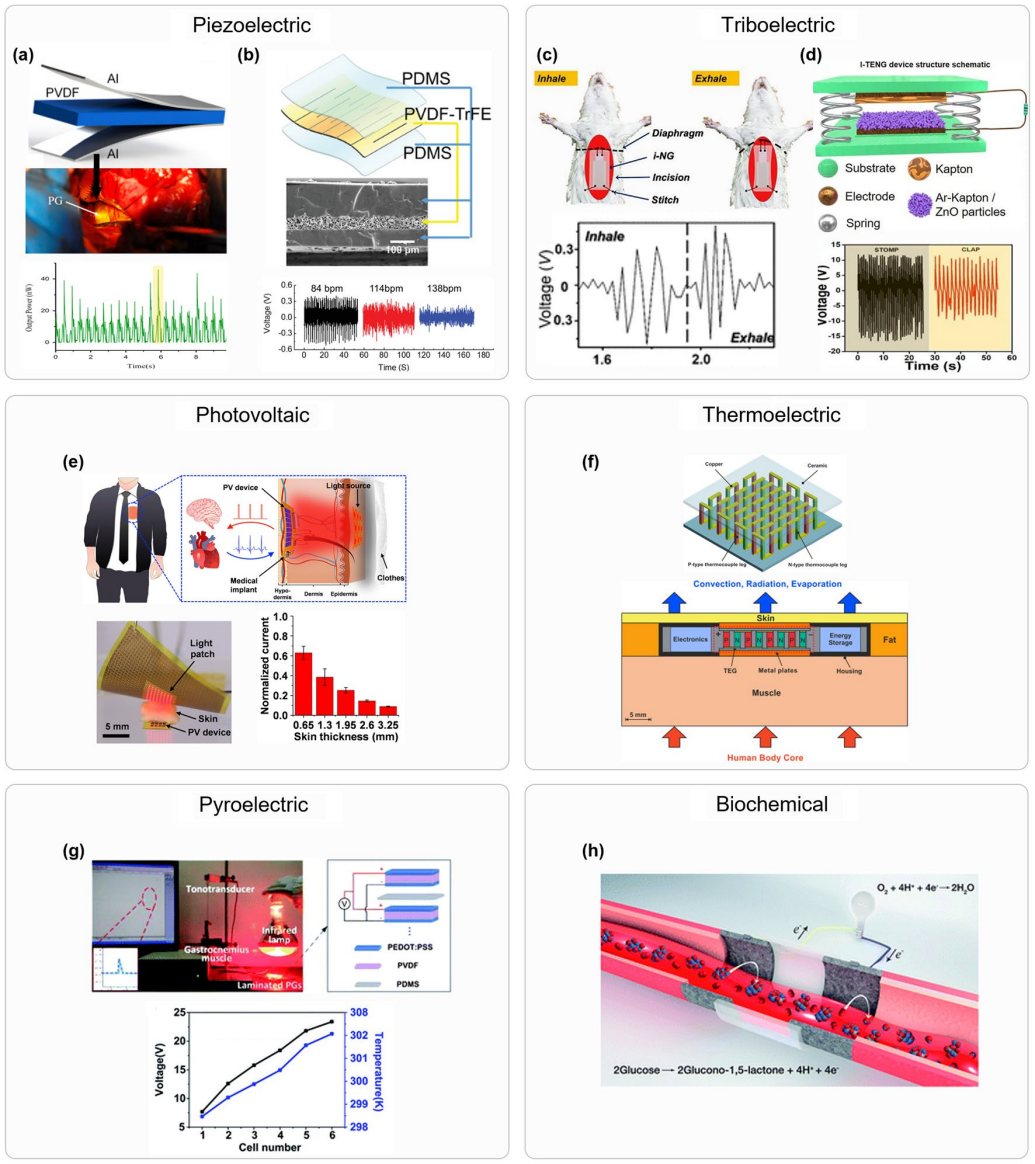
Energy harvesting techniques. Piezoelectric (**a, b**): **a** illustration of the piezoelectric generator to collect energy from the pulse in the aorta, its implantation and graph of the obtained power. Reproduced with permission [36]. Copyright 2015, Elsevier. **b** Diagram of piezoelectric generator based in a Kirigami design, image of the assembled device, and voltage waveforms. Reproduced with permission [38]. Copyright 2021, Wiley–VCH. Triboelectric (**c, d**):** c** diagram of the working principle of a triboelectric generator that harvests energy from breathing and a graph of the voltage collected by such device. Reproduced with permission [39]. Copyright 2018, ACS. **d** Schematic of the design of the ion-implanted TENG and its voltage output. Reproduced with permission [40]. Copyright 2021, Elsevier. Photovoltaic: **e** diagram of possible utilization of the implanted PV device coupled with a micro-LED wearable device, and a graph of the obtained current at different implantation depths. Reproduced with creative commons license [45]. Thermoelectric: **f** render of thermoelectric generator and diagram of the device implanted below the skin. Reproduced with permission [106]. Copyright 2022, Elsevier. Pyroelectric: **g** image of the device under test conditions and diagram of multicell connection with a graph showing the voltage obtained. Reproduced with creative commons license [82]. Biochemical: **h** diagram of implanted biofuel cell. Reproduced with Creative Commons License [83]

There are multiple sources of kinematic energy produced by the human body. The contraction and extension of multiple different muscles can be explored to produce energy. Moreover, the pressure exerted by the body can also be used by energy harvesters. Different approaches can be used to transform kinetic energy into electrical energy, such as the use of piezoelectric generators, triboelectric generators, or electromagnetic generators. In the case of piezoelectric generators, mechanical vibrations inside the body are used to induce the piezoelectric effect in the harvester. Zhang et al. [37] used a PVDF strip wrapped around the ascending aorta to transform its pulsatile expansion into electrical energy, generating up to 681 nW. The device and a graph of the output power are shown in Fig. 4a. Researchers have also been investigating Kirigami-based designs for piezoelectric generators to achieve better flexibility and conformity with organs. In Ref. [38], a Kirigami-based stretchable piezoelectric generator was proposed to generate up to 228 nW, improving current designs using an intersegment electrode design. Xu et al. obtained up to 2.4 µW from another Kirigami-based device by harvesting the energy in the motion of the heart [39]. A schematic of the device and a graph of its output voltage are shown in Fig. 4b. The group also conducted an in vivo test that was able to generate voltages up to 0.7 V when implanted into the heart of a porcine. The use of triboelectric nanogenerators (TENG) is another approach to harvesting energy from motion produced by the body based on the triboelectric effect. The triboelectric effect is based on the charge build-up in the surfaces of materials when they come into contact with pressing or friction. When the materials are separated, some of those charges will remain, creating an electric potential between them, as the charges have opposite signs [105]. Li et al. [40] created a device to harvest energy from breathing, testing it in vivo with rats’ models with an average power of 0.12 µW. The device, shown in Fig. 4c, was able to convert the low-frequency breathing movement into a steady 2.2 V output thanks to the usage of a rectifier and a capacitor. TENG can have its capabilities enhanced with the implantations of ions or particles on the surface of the materials. Sahu et al. [41] implanted argon ions into Kapton and used ZnO particles to improve the power generated by a TENG device. A schematic of the device and voltage waveforms is shown in Fig. 4d. In this study, the TENG device with ZnO particles was shown to perform better than the argon-implanted Kapton and the pure Kapton devices. The most common way to convert kinetic energy into electrical energy is the use of electromagnetic induction. In this method, motion is used to move a magnet inside a copper coil, creating a change in its magnetic flux, creating a potential. Due to the need of a moving part besides the coil, this technique has not been explored a lot in the implantable device literature. But electromagnetic induction has been proved to successfully collect power from the heart motion [42]. In this study, a stack of permanent magnets was suspended between two flexures and positioned inside a copper coil. This device was implanted inside a pig’s right ventricular cavity, generating a maximum of 1.7 µW at 160 bpm.

##### Photovoltaic Energy

There has been much interest in photovoltaic energy generation due to the global increase in investment in green energy. This modality of electrical energy generation has also been investigated to power implantable medical devices. Those devices can easily harvest energy from externally to the body as there are multiple light sources available in most environments. However, photovoltaic generators cannot be implanted deep into the body due to the absorption of light by biological tissue. Therefore, those devices are usually utilized to power subcutaneous implants, but they still suffer from low power capabilities, as the skin reflects and absorbs much of the light it receives. This being said, the feasibility of long-term usage has already been validated [43]. In this study, volunteers wore a device that continuously monitored the power output of subcutaneous solar cells throughout their daily routine for a week during the summer, fall, and winter. The mean power density obtained throughout the study was 19 µW cm^−2^, a high value for energy harvesting, showing promising results. This method of energy harvesting utilizes the photovoltaic effect to convert energy contained in light to electrical energy that is used for powering the implants. When lights go through a semiconductor material, photons will excite electrons in the valence band, making them move to the conduction band. These electrons become accelerated in the rectifying junction, creating a potential and a current. Wu et al. [44] created a flexible GaAs photovoltaic cell when placed under 3 mm of porcine skin generated up to 1.35 mW with sunlight and 0.12 mW with a lamp. Infrared energy can also be harvested by PV cells. Moon et al. [45] compared using GaAs and Silicon PV cells to harvest energy from infrared radiation in different places in a mouse model—Photovoltaic infrared subcutaneous energy harvesting. The GaAs cell was able to reach a power density of up to 12.24 µW mm^−2^, while the silicon cell reached 7.75 µW mm^−2^. Kim et al. [46] demonstrated a subcutaneous photovoltaic device illustrated in Fig. 4e. The device has an area of 11.1 mm^2^ and is capable of harvesting up to 74.1 µW cm^−2^. In this study, a flexible GaAs PV device was implanted in a mouse model and an external flexible red micro-LED array was used to stimulate the implant. The red light was chosen due to its deeper tissue penetration, allowing the device to electrically stimulate the mouse heart.

##### Thermal Energy

Thermoelectric generators have also been an area of intense research interest in the previous few years. These kinds of devices are able to transform the thermal energy found in a temperature gradient into electrical energy through the use of semiconductor and conductor material properties. This is done through the use of the Seebeck effect, in which charge carriers from the hot side diffuse to the cold end, creating an electrical potential between those two ends [107]. Due to the need for a temperature gradient for these kinds of devices to work, thermoelectric generators (TEG) are more well suited for wearable devices, as they will be in contact with the cold outside environment and the body. These kinds of devices could also be used in subdermal implants to power implantable medical devices (IMDs) potentially. A comprehensive review of developments in the field of TEGs focused on medical applications was published in Ref. [108] and delineates steps to improve their efficiency.

The suitability of the usage of TEG devices to power IMDs was investigated by Yang et al. back in 2007 [81]. In in vitro experiments, using a copper plate at 310 K to simulate body heat and a room temperature of 291 K, a 0.5 K temperature difference between a commercial TEG’s hot and cold sides was observed at equilibrium, generating a voltage of around 3.3 mV. A 1.1 K temperature difference was obtained with the use of icepacks and cold water on the skin. In vivo studies were also performed, obtaining a temperature difference of 1.3 and 5.5 K with the use of ice bags, obtaining a voltage of 25 mV. The power generated by the device was not measured as the authors considered it insufficient, but they estimated that more advanced devices, such as the one presented in Ref. [109], could potentially generate power between 14.4 µW and 2.88 mW. More recent studies found through the use of simulations that up to 100 µW can be obtained with the implantation in the abdomen [106]. This study points out different optimizations can be made to improve the device's power generation, but only simulations were conducted, with no physical tests.

Another way to generate power from a thermal source is using pyroelectric generators. These kinds of devices can transform temperature fluctuations into electrical energy, as these fluctuations create a change in polarization in the pyroelectric material. These materials are a subclass of dielectrics in which polar symmetry and spontaneous polarization are observed without an electric field [110]. An extensive review of these kinds of devices and its developments was published in Ref. [110], but no implantable applications were shown. In Ref. [82], a soft pyroelectric generator designed to be implanted into the body was shown. When being illuminated by an infrared source, a multicell device could provide 2.3 V to power an LCD screen and electrically stimulate muscle. However, no biological tissue was placed between the power source and the device.

##### Biochemical Energy

The use of biofuel cells (BFCs) is another way to harvest energy from living organisms. Through the use of redox reactions of energy-carrying substances, enzymatic fuel cells can generate electrical energy that can be used to power IMDs [111]. In this type of biofuel cells, the biofuel is used in the anode, an oxidant is used on the cathode, and oxidoreductases are used as catalysts, enabling the redox reaction. The reaction will generate an electron flow between the anode and cathode, generating electrical energy. A review of enzymatic biofuel cells and their research progress is present in [112].

This energy generation technique was shown ex vivo using human blood in a vein replica by Pankratov et al. [83]. In this study, a model of a vein with graphite electrodes was connected to veins in the arm of human subjects and was able to obtain a 0.31 V potential and 0.74 µW maximum power output. This study proves the capability of enzymatic fuel cells to power IMDs. In Ref. [84], an enzymatic biofuel cell was implanted into a bird to power a brain stimulator device and send the power generation data through Zigbee. The BFC was able to generate an average power of 0.048 mW.

### Wireless Communication

**Fig. 5:**
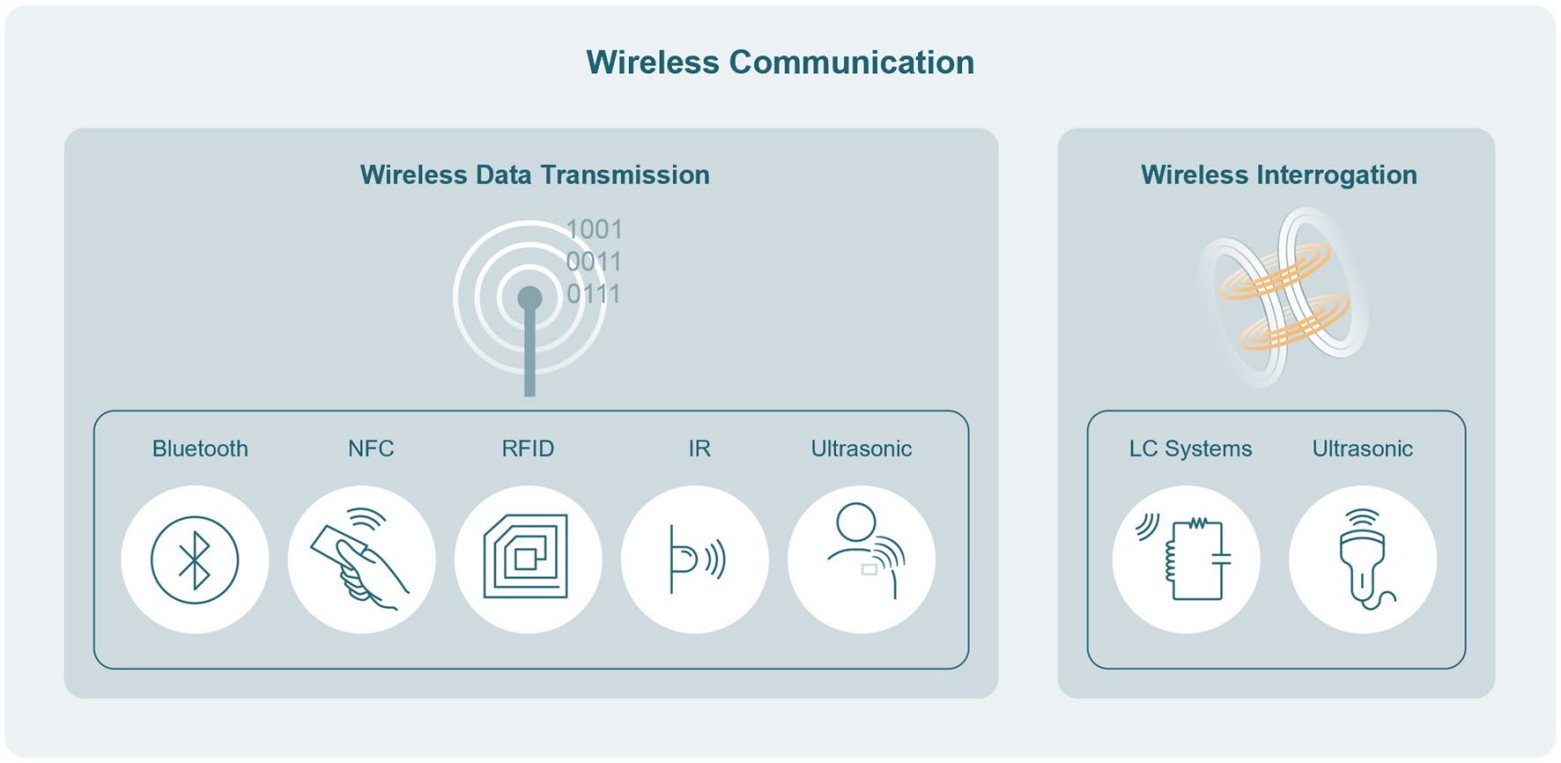
Diagram of wireless communication techniques: wireless data transmission and wireless interrogation

The use of wires in implants can lead to infection, and if the data are only stored in the device, subsequent surgery will be needed to have access to it. Therefore, to collect the data safely measured by implantable biosensors, the data retrieval needs to be realized wirelessly. To do so, the device can transmit its data or have its data encoded in its physical state, which can be interrogated externally. To achieve a wireless communication strategy for the implantable device, there are two main categories: wireless data transmission and wireless interrogation. These categories include multiple different techniques, as illustrated in Fig. 5. Careful consideration of the requirements of each application, such as the available power, the number of devices, and the implantation depth, will be needed when choosing the optimal way to retrieve the data.

#### Wireless Data Transmission

As we delve into the intricacies of data collection and transmission in implantable devices, it is crucial to understand the wireless aspect. In wireless data transmission, the device must collect, encode, and send data to an external device. Utilizing established communication protocols such as Bluetooth or Wi-Fi enhance compatibility with existing devices. However, the signal frequencies that consist of those protocols, around 2.4 GHz, are significantly attenuated by the body. From the most established communication protocols, one of the better suited to implantable devices is Bluetooth low energy (BLE). This is because of the reduced power consumption, consuming between 0.01 and 0.5 W, but if the throughput is reduced, the average power can go as low as a few microwatts [113]. It is also compatible with most existing consumer devices and most notably with smartphones. In Ref. [44], a solar energy harvester powered a BLE module, consuming, on average 400 uA while active and 7 uA while sleeping. Zhong et al. developed a bladder pressure monitoring that uses the BLE protocol and is powered by magnetic coupling [114]. Another established set of communication protocols is near-field communication (NFC). This method uses inductive coupling to create a link between devices up to 4 cm away and can both transmit data back and forth and transmit power. It operates at a frequency of 13.56 MHz, which is less absorbed by biological tissue than 2.4 GHz. Moreover, some protocols are also compatible with smartphones, as they use NFC for payment services. The feasibility of monitoring NFC implantable devices with a smartphone was investigated in Ref. [115]. In this study, a 3-coil system is also proposed to increase the readout distance. To increase the NFC capability in a weak coupling regime, Gong et al. used two different carrier frequencies and binary phase shift keying instead of load shifting keying [116]. Radio-frequency identification (RFID) is another common communication protocol that has been used in a multitude of different areas and has also been explored in the implantable medical device field, allowing for the creation of passive and active devices. Passive RFID is an application of backscattering in which the signal is received, modulated, and reflected to the reader. This technology and antenna design for RFID are discussed in depth in Ref. [117]. One benefit of this method is the long readout distance. In Ref. [118], an antenna measuring $\uppi \hspace{0.17em}$× (6)^2^ × 1.27 mm^3^ was shown to enable readout distances as long as 87 cm. Other ways to transmit data without the use of RF include the use of IR or ultrasound. In Ref. [3], an IR led is modulated using ON/OFF keying using a carrier frequency of 57 kHz, allowing for a data rate of 27 Hz. But most of the transmitted light is absorbed by the body, with the transmission in the in vivo study varying from 53% in certain species of rats to as low as 1% in others. In the case of data transmission using ultrasound, Weber et al. developed a device that uses a piezoelectric transducer that can transmit data at a depth of 12 cm, with a device that measures 1.7 × 2.3 × 7.8 mm^3^, showing great promise for deep implants [119]. When there are multiple sensors implanted in the body, and all of them need to be sampled simultaneously, new challenges arise as the data transmitted by each device needs to be identified and cannot overwrite each other. In Ref. [120], an RF backscattering protocol that can transmit up to 10 Mbps using time-domain multiple access (TDMA) protocols is demonstrated. This network could support up to 1000 channels and was demonstrated in vivo with the use of 56-neurograin array implant. To improve on some TDMA shortfalls, such as interference between power and data links and assignment of time to multiple devices and increase the number of possible nodes in the network, Feng et al. [121] proposed using code-division multiple access (CDMA). CDMA allows multiple nodes to transmit data in the same channel as each user modulates their signal according to their code. Due to the limitations in size and power available for implantable devices, data encryption and security are usually nonexistent in most proposed devices. But to achieve clinically applicable devices, meeting medical data security requirements is essential. In Ref. [122], a device that performs in-situ encryption before sending it through NFC is demonstrated. Moreover, the data collected are also protected using blockchain technologies.

#### Wireless Interrogation

To communicate the data acquired by the biosensor to the external world, the device does not necessarily need to transmit the data. Devices can also change their physical state in a way that can be interrogated externally. In the case of LC systems, a change in the sensor value can create a change in the resonance frequency of the device. This can be observed by an external device such as a vector network analyzer. A device can also change its physical shape or its acoustic absorption in response to changes in the target signal. These changes can be interrogated by imaging the device with an ultrasound transducer. As those devices do not have to consume power to share their data, most wireless interrogated devices are passive.

##### LC Systems

LC systems consist of a capacitor connected to an inductor, making it resonate at a certain frequency. When the inductor is coupled to an external inductor, the transmission efficiency will be maximum when the frequency of the signal going through the external inductor is equal to the resonance frequency. By performing a frequency sweep and monitoring either the reflection coefficient or the impedance, one can look for a peak to find the resonance frequency of the implant externally. The resonance frequency can be related to the signal of interest using a capacitive or inductive sensor, with the first option being the most used one. The first implantable LC sensor was proposed by Collins et al. in 1963 [123] and consisted of a capacitive pressure sensor connected to a coil to be implanted in the eye. Since then, multiple studies have been published utilizing this technique, as it requires few components and is completely passive. The main challenges with LC systems are the weekly coupling between the coils due to the limited space for the implanted coil and the distance between the implant and the external coil. Moreover, misalignment between the coils can also make the coupling weaker. To solve these issues, good coil design is fundamental. The more traditional coil designs are circular and square planar coils. Optimization of square coils is shown in [124, 125] and for round coils in [50, 51]. Different designs besides square and circular coils were also investigated. In Ref. [126], circular, square, octagonal, and 16-side coils were compared. This study concluded that the square coil gives the highest inductance and, thus, the highest quality factor for the same size. Huang et al. [127] compared 2D and 3D structures, comparing a planar inductor with a solenoid inductor. It was concluded that 3D structures can achieve higher quality factors and the magnetic field outside of the axis is smaller than in 2D structures, reducing the interference between inductors. Another way to increase the quality factor of the device and its readout distance is to decrease the resistance of the coil. In Ref. [8], an investigation on how the thickness of the coil, and thus its resistance, impacts the wireless performance of the device was carried out. The use of a ferrite core can also increase the magnetic coupling of the coils [11, 128]. This occurs because the coil's magnetic permeability will increase, and thus, its inductance will also increase. To increase the wireless capabilities of LC systems, new reading systems have also been proposed. The exploration of exceptional points (EPs) in parity-time symmetric systems has recently been proposed to extend the wireless interrogation of LC systems well beyond their current capabilities. The general condition of PT symmetry is achieved when the gain and loss parameters are balanced, and the impedance of the reader and the sensor, when multiplied by *i*, are complex conjugates of each other [129]. Due to the geometrical limitations of the implant, this is rarely achieved without severely reducing the reader size and downgrading its performance. To circumvent this problem, Chen et al. [129] proposed a PTX-symmetric system, taking into account the difference in size between the reader and the sensor systems, and showed it to greatly enhance the quality factor of the system. The PTX symmetry was also demonstrated to be robust against coil misalignment, improving the reliability of the system [52]. Other uses of EPs in PT symmetry were shown to increase the readout distance of the LC sensors by 3.2 times [53] to 4 times [130]. The use of repeaters has also been proposed to extend the range of LC systems. In Ref. [131], the use of a repeater was shown to increase the readout distance by 180% without sensitivity decay. This was achieved by using a coefficient matrix to derive the two peak frequencies for the sensor–repeater system. Repeaters using metamaterials were also shown to increase the readout distance by 1.8 times [132]. Dong et al. [133] proposed a repeater that can change its resonance frequency to match the one in the sensor, achieving a distance of 3.18 times as long as without a repeater. Another way to increase the readout distance is the use of impedance matching, which was shown to improve the readout distance as much as 1.5 times by using the topology proposed in Ref. [134].

##### Ultrasound

Another way to wirelessly interrogate a sensor is with the use of ultrasound to measure the device’s shape or its acoustic characteristics. This is usually obtained using stretchable materials, fluids, or microbubbles. These types of devices are usually used for measuring pressure and strain in different organs. The advantages of these techniques again LC systems are the lower absorption of ultrasound by biological tissue, better efficiency when dealing with miniaturized devices, cheaper readout system, and less complex fabrication procedure [48]. In the cases of pressure sensors, their working principle can be divided into two main categories: microbubbles and fluidic-based systems [48]. For the microbubble technique, microbubbles filled with gas are injected into the tissue. Pressure changes can lead to either the disappearance of microbubbles, a shift in the harmonics, or the amplitude of scattered echo, with the last approach being the most promising [47]. Li et al. [47] researched the effect of different acoustic parameters on the sensitivity of this approach. In the fluidic-based approach, the device is constructed in such a way that external pressure changes the fluid level inside microchannels, which can be observed via ultrasound imaging. This technique offers higher sensitivity and is better suited for long-term pressure monitoring than microbubbles. In Ref. [48], a microfluidic device made of PDMS is presented, having a sensitivity of 42 kPa mm^−1^ of displacement. To measure strain, a flexible material with an acoustic impedance that is different from the body can be imaged, and the difference in its shape can be calculated. In Ref. [49], a stretchable hydrogel was mixed with ZnO particles, which offer a high acoustic impedance, allowing it to be imaged by the use of ultrasound. The implantation of ZnO also had the effect of increasing the hydrogel strength and elasticity. When the device is stretched, its cross-sectional area is reduced with a rate of 0.24% per 1% strain change, and when its cross section is measured with ultrasound, its strain can be calculated.

## Materials

### Biocompatible Materials

To be considered biocompatible, a material must not cause an unacceptable degree of harm to the host. Biocompatibility is often assessed by evaluating a material’s cytotoxicity or carcinogenicity [135]. The toxicity and carcinogenicity of material are evaluated using in vitro cytotoxicity assays, while in vivo implantations are used to evaluate more complex host reactions. However, toxicity alone does not determine biocompatibility. An implantable sensor must also be able to match changes in morphology at the implantation site to minimize changes or damage to the surrounding tissue. Davis’s law states that soft tissues will heal and structurally adapt according to the mechanical stresses imposed upon them [136, 137]. By exceeding the mechanical demands of the native tissue, high-modulus materials may cause excessive stress and multimodal deformation to the underlying tissue, resulting in the degradation of the surrounding tissue [138]. This tissue damage can exacerbate unwelcome foreign body responses [135, 139]; as cells attempt to remove the detected foreign body, they release cytotoxic and neurotoxic biological factors into the peri-implant space, resulting in local cell death [140], undesirable chronic inflammation and tissue degeneration, or fibrosis and encapsulation of the desired implant that renders the sensor unusable [138]. In the development of biomaterials, the challenge lies not in developing biocompatible materials but in combining biological and mechanical compatibility. As a result, physical softness is of utmost importance, with toughness, elasticity, and flexural rigidity some of many characteristics that determine softness [135, 139, 141, 142]. The biggest challenge in developing mechanically compatible biomaterials is requiring highly soft and compliant materials. Traditional sensing materials are often too rigid to be mechanically compliant with soft tissues. In contrast, native soft tissue typically has Young’s moduli in the range of 0.1 kPa to 1 MPa [143], and gold film and bulk silicon have moduli of 69 and 130–188 GPa, respectively [144, 145]. As a result, several different approaches have been taken to develop both intrinsically and extrinsically compliant materials, using nanoscale and hierarchical structures and controlled mechanical buckling [146]. Different materials have properties that make them ideal for different applications. Strong electrical and mechanical properties of metals and carbon-based materials have led to their usage in electrodes and interconnects, while due to their relatively soft, conformable, and tuneable nature, polymers are often used as substrates and biofluid barriers. Piezoelectric polymers have also been used as strain sensors. In cases where multiple properties are desired, composites have been developed to combine seemingly contradictory characteristics into a single material. In this section, novel biocompatible materials are described, with a focus on biocompatible metals, carbon-based materials, polymers, and composites. Table 2 shows comprehensive information about various categorized biocompatible materials for implantable electronics by comparing core features such as cytotoxicity, mechanical modulus, and stretchability.

**Table 2:** Comparison of biocompatible materials for developing implantable electronics

Type	Material	Cytotoxicity	Mechanical properties		Electrical properties	Application	References
			Stretchability (%)	Modulus (MPa)	Resistivity		
Metal	Buckled Au nanotrough network	–	300	70,000	≈ 10 Ω sq^−1^	Flexible transparent electrodes	[147]
	Microhole gold thin film	Cell growth during 96 h incubation	94	–	6e^−5^ Ω cm	Biosensing Electrode	[148]
	v-Au NWs	–	800	–	0.00625 Ω cm	Glucose and strain sensor	[149]
	EGaInSn liquid metal	–	160	–	2.16e^−4^ Ω cm	Flexible strain sensor	[150]
	Biphasic film of liquid gallium onto gold	–	400	–	0.5 Ω sq^−1^	Movement strain sensor	[151]
Carbon	Helical twisted carbon nanotube fiber bundles	95% cell viability after 5 days	7	118.9	–	Implanted electrochemical biomarker sensor	[152]
	Coiled carbon nanotube (CNT) yarn	> 90% cell viability after 7 days	> 30	–	–	Gastric deformation sensor	[13]
	Carbon nanotube (CNT) fibers	Cell numbers maintained for 72 h	–	–	–	Fiber power generator	[153]
	Binder-free Ox-SWCNT buckypaper	In vivo: 100% cell viability after 3d	–	–	3.47 Ω cm^−2^	Supercapacitor energy storage system	[154]
	Prussian, blue-doped carbon ink	74.1% cell viability	–	–	–	Implanted continuous blood glucose monitoring sensor	[155]
	Carbon nanotube array	Similar mean fluorescence intensities to control after 4 weeks	245	–	–	ECoG and ion-selective electrode	[156]
Polymers	NaCl/SA/PAM DN hydrogels	–	3120	0.05	1000 Ω cm	Epidermal strain sensor	[157]
	QCS-AMP/PAAm hydrogels	Same cell viability after 96 h	1731	0.0229	35.714 Ω cm	Epidermal strain sensor	[158]
	HK/PVA/NaCl hydrogels	No tissue inflammation	–	0.11	12.048 Ω cm	Epidermal strain sensor	[159]
	PVA/Amy/NaCl hydrogel	98.7% cell viability after 24 h	500	0.7	29.412 Ω cm	Epidermal strain sensor	[160]
	poly(HEAA-*co*-SBAA)/PEDOT:PSS hydrogels	90% cell viability after 48 h incubation	~ 5000	0.05–0.18	160 Ω cm	Epidermal strain sensor	[161]
	Alginate hydrogel iontronic fibers	Similar cell viability to control after 5 days	77–227.7	0.1–2.6	0.05–18.52 Ω m	Physio electrical modulator	[162]
	Reconstituted silkworm silk fibroin/polyethylene oxide polymer film	–	175–1200	0.06–18 (hydrate)	–	–	[163]
Composite	Ag–Au core–sheath nanowire composite	30% fibrotic area 3 weeks after implantation	266	–	1.3774e^−5^ Ω cm	Electrophysiological signal measuring patch	[164]
	PDA–clay–PSBMA hydrogels	Inflammatory responses disappeared at 7 days	700–900	–	4651.2 Ω cm	Organ motion sensor	[14]
	(GF/PAM/CA) strain sensor	No tissue damage and inflammatory lesions	600	0.0137–0.090	Initial resistance of ∼ 20 Ω	Epidermal strain sensor	[165]
	Hydrogel hybrid probes	Reduced foreign body response	–	0.0165	–	Probes for neural electrophysiological sensing	[166]
	Liquid metal fiber mat	95% cell viability after 24 h	> 1800	~ 0.1	5.5556e^−5^ Ω cm	Electrocardiography (ECG) sensor, a sweat sensor	[167]
	Core/shell poly(vinylidene difluoride) (PVDF)/dopamine (DA) nanofibers	Over 70% cell viability after 72 h	–	–	–	Piezoelectric pressure and force sensor for organ motion	[168]

#### Metal-Based Materials

**Fig. 6:**
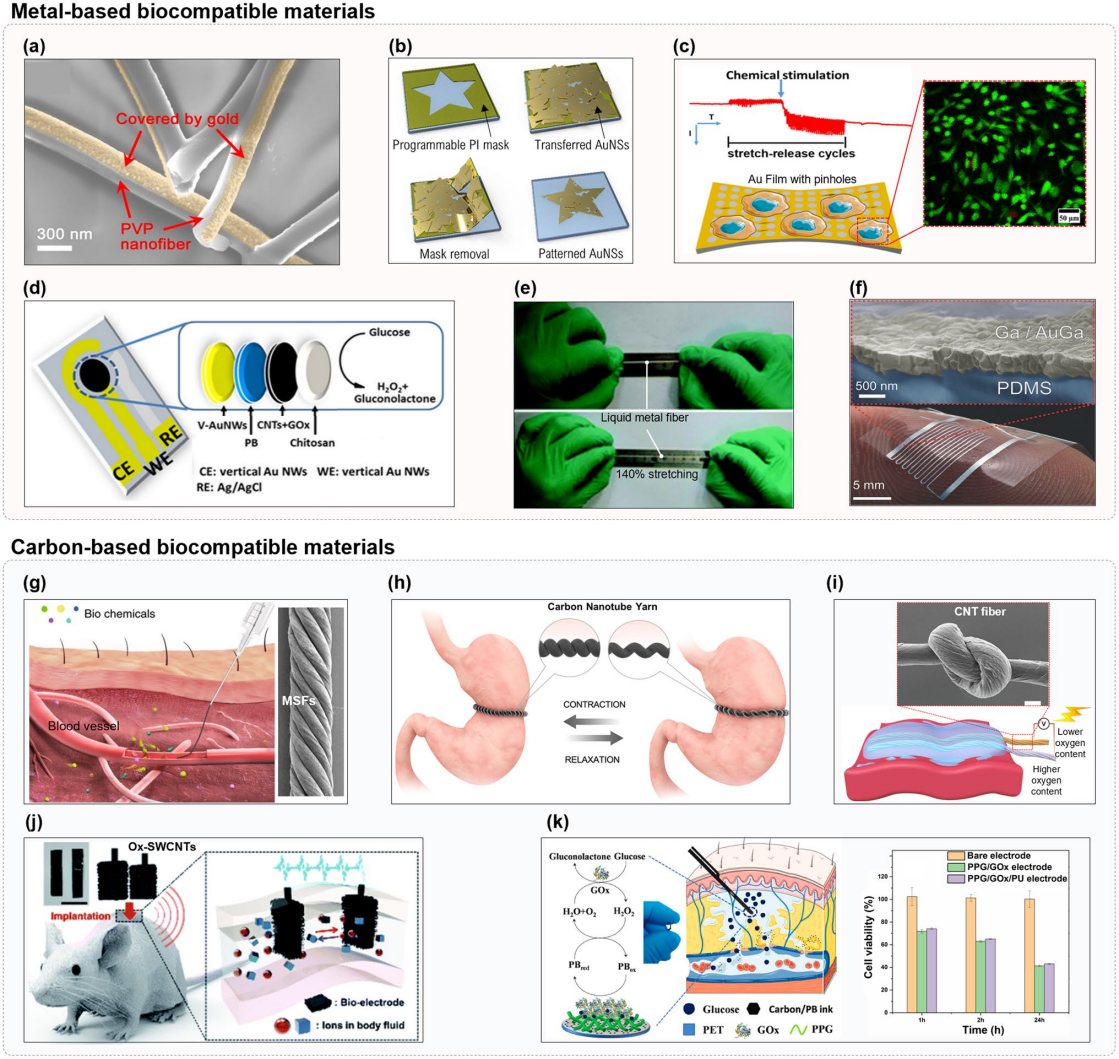
Recent progress of the biocompatible materials for implantable devices: Metal-based materials (**a**–**f**), carbon-based materials (**g**–**k**). **a** Reproduced with permission [147] Copyright 2017, Wiley-VCH. **b** Reproduced with permission [169] Copyright 2020, Elsevier. **c** Reproduced with permission [148] Copyright 2020, ACS. **d** Reproduced with permission [170] Copyright 2019, ACS. **e** Reproduced with permission [150] Copyright 2017, RSC. **f** Reproduced with permission [151] Copyright 2016, Wiley-VCH. **g** Reproduced with permission [152] Copyright 2020, Springer Nature. **h** Reproduced with permission [13] Copyright 2019, ACS. **i** Reproduced with permission [153] Copyright 2022, Elsevier. **j** Reproduced with permission [154] Copyright 2022, RSC. **k** Reproduced with permission [155] Copyright 2022, Elsevier

Metals, with their robust strength and electrical conductivity, are often used as electrodes and interconnects. However, most metals are not suitable for use in implantable; metals and their corrosion or leaching products can be cytotoxic, while bulk metal often does not have the ability to conform sufficiently to implantation sites [139]. Gold is popularly used in biosignal sensors due to its high conductivity and low cytotoxicity; however, gold, like other metals, is intrinsically inelastic, requiring the use of nanowire networks or structural designs to impart elasticity. While many of the materials discussed in this section were developed as on-skin wearable electronics, they were all developed using biocompatible materials that would be sufficient for implantable use. This can be seen in Huang et al.’ study, where gold is used in a buckled nanotrough network to impart extrinsic stretchability and flexibility to otherwise inelastic gold on-skin electrodes [147] (Fig. 6a). To make the buckled Au nanotrough network, an interconnected Au nanofiber network was fabricated and transferred onto pre-strained PDMS substrate. Upon release, the PDMS substrate causes buckling in the nanofiber network and creates wavy microstructures (*R*_0_ = 29.4 Ω sq^−1^) that were able to reach 100% strain with only a 6% increase in resistance, and an optical transmittance of up to 91%. Jeong et al. used a simpler approach to imbue elasticity to gold electrodes, by heat-pressing 2D gold nanosheets onto a PDMS substrate to measure electrocardiogram (ECG) and electromyograph (EMG) signals [169] (Fig. 6b). Through this process, the electrodes maintain electrical conductivity while stretched, with a sheet resistance *R*_s_ of as low as 5 Ω sq^−1^ at up to 50% strain. Another simple method developed by Ling et al. [148] used highly ordered pinholes to measure and monitor hydrogen peroxide (H_2_O_2_) released from living cells (Fig. 6c). By creating pinhole microstructures in the thin gold films, stresses caused by tensile strain are redistributed around each hole, preventing the emergence of catastrophic channel cracks. As a result, the holey gold film was able to achieve strains up to 94% before loss of conductivity compared to only 4% in non-structured films. While 2D thin films are commonly used in stretchable electronics, the use of 1D nanomaterials have also proved attractive as a biocompatible material. In a paper by Zhai et al., vertically aligned Enokitake-like gold nanowires (v-Au NWs) were used to develop on-skin wearable pressure sensors. The developed sensors were both biocompatible and highly stretchable up to 800% strain without loss of conductivity [170] (Fig. 6d). While the microstructuring of thin metal films imparts good stretchability, it also results in reduced conductivity. Liquid metals and their ability to flow and deform while maintaining good conductivity opens a new avenue for stretchable metals [171]. Gallium (Ga) and its alloys have been the focus of recent liquid metal research, due to their comparatively low toxicity, vapor pressure, and viscosity [171, 172]. In Wu et al. [150], a liquid metal fiber sensor was developed that imitated the structure of blood vessels to measure human body motion. The sensor was constructed using a superelastic biocompatible fiber channel injected with a low-toxicity liquid metal (EGaInSn) (Fig. 6e). When strained, the liquid metal deformed with the stretched sensor, and could withstand strains of up to 140% without loss of conductivity. However, liquid metals have their drawbacks, with common patterning techniques unable to achieve high-resolution batch processing across larger surface areas. To combine the batch processing capabilities of metallic thin films and the conductivity of liquid metals, Hirsch et al. developed a biphasic gold–gallium thin film conductor using gold thin films and liquid gallium. This allowed them to create an epidermal flexion sensor with a low sheet resistance of 0.5 Ω sq^−1^ and capable of withstanding uniaxial stretching up to five times its length [151] (Fig. 6f). Fabrication was conducted in two steps; a PDMS substrate was first sputtered with gold to provide an alloying thin film, onto which liquid gallium was evaporated on.

#### Carbon-Based Materials

While concerns regarding the cytotoxicity of carbon nanotubes exist, they remain a popular material for use in implantable sensors due to their attractive properties, such as their high mechanical stability and electrical properties [173–175]. Several studies have assessed the cytotoxicity of carbon nanotubes (CNTs) [176–179], and it was found that the biocompatibility of CNTs is tuneable, as functionalization and shortening of CNTs can be used to reduce cytotoxicity to levels safe for implantation [178, 180–183]. Yang et al. used carbon nanotube arrays to develop a flexible multi-functional electrode (FME) capable of simultaneously recording ECoG signals as well as extracellular ions on the surface of the cerebral cortex [156]. Vertically aligned carbon nanotube arrays (CNTAs) were patterned and formed on silicon wafers through chemical vapor deposition and transferred to a flexible PDMS substrate to form the desired electrodes. The ECoG electrodes developed were found to have a high specific capacitance of 1.51 mF cm^−2^, much higher than that of standard gold electrodes (0.11 mF cm^−2^) and were able to maintain their impedance while immersed in 0.01 m phosphate-buffered saline (PBS) and artificial cerebrospinal fluid (aCSF) for 14 days. By building off of this CNTA, ion-specific electrodes were further developed. Three different ion-selective membranes were dip-coated onto the CNTAs, creating electrodes sensitive to Ca^2+^, K^+^, and Na^+^ with sensitivities measuring 28.86, 48.38, and 52.28 mV dec^−1^, respectively. In Wang et al., carbon nanotubes were twisted into hierarchical and helical fiber bundles that mimicked muscle filaments [152] (Fig. 6g). Fillers such as Pt nanoparticles were used to form single-ply sensing fibers (SSFs), which could be twisted with other SSFs to form multi-ply sensing fibers (MSFs) for simultaneous detection of different biomarkers. Due to the CNTs’ low bending stiffness, their small size, and hierarchical structure, the sensor can match the bending stiffness of surrounding tissue, reducing mechanical mismatch. Jang et al. [13] took a similar approach of Wang et al., using coiled carbon nanotube yarn that operates as energy harvesters or self-powered strain sensors for real-time evaluation of gastric motility in an aqueous environment without need for encapsulation (Fig. 6h). Sheets of forest-drawn multi-walled carbon nanotubes (MWNTs) were spun into high-strength yarns. Twisting or stretching of the CNT yarn causes the rearrangement of electrical charges due to surrounding ion movement and generates an electrical voltage. A simpler flexible fiber power generator was developed by He et al. [153] that was fabricated from two CNT fibers containing modified oxygen-containing groups aligned in parallel (Fig. 6i). When immersed in biofluids, a voltage and electricity is generated, with a maximal output power density of 569.4 mW g^−1^ when immersed in 2.5 M HCl. A different approach to CNTs were used by Chae et al., who used oxidized single-walled carbon nanotubes (Ox-SWCNTs) in the form of a binder-free buckypaper as a flexible supercapacitor (SC) cell for use as a high-energy implantable energy storage system [154] (Fig. 6j). Its flexible, strong network between constituent SWCNTs imbues greater biostability, and electrochemical oxidation was used to further improve biocompatibility. Other forms of carbon from CNTs have also been investigated for sensor use; Jin et al. [155] used an easily prepared graphene oxide carbon ink to fabricate a wearable, screen-printed continuous glucose monitoring sensor (Fig. 6k). Carbon ink has often been used for screen-printed sensors due to its high conductivity, low cost, and excellent mechanical properties. In this study, the carbon ink was doped with Prussian blue (PB) to act as an electron transfer mediator to allow detection of glucose at low applied potentials.

#### Polymer-Based Materials

**Fig. 7:**
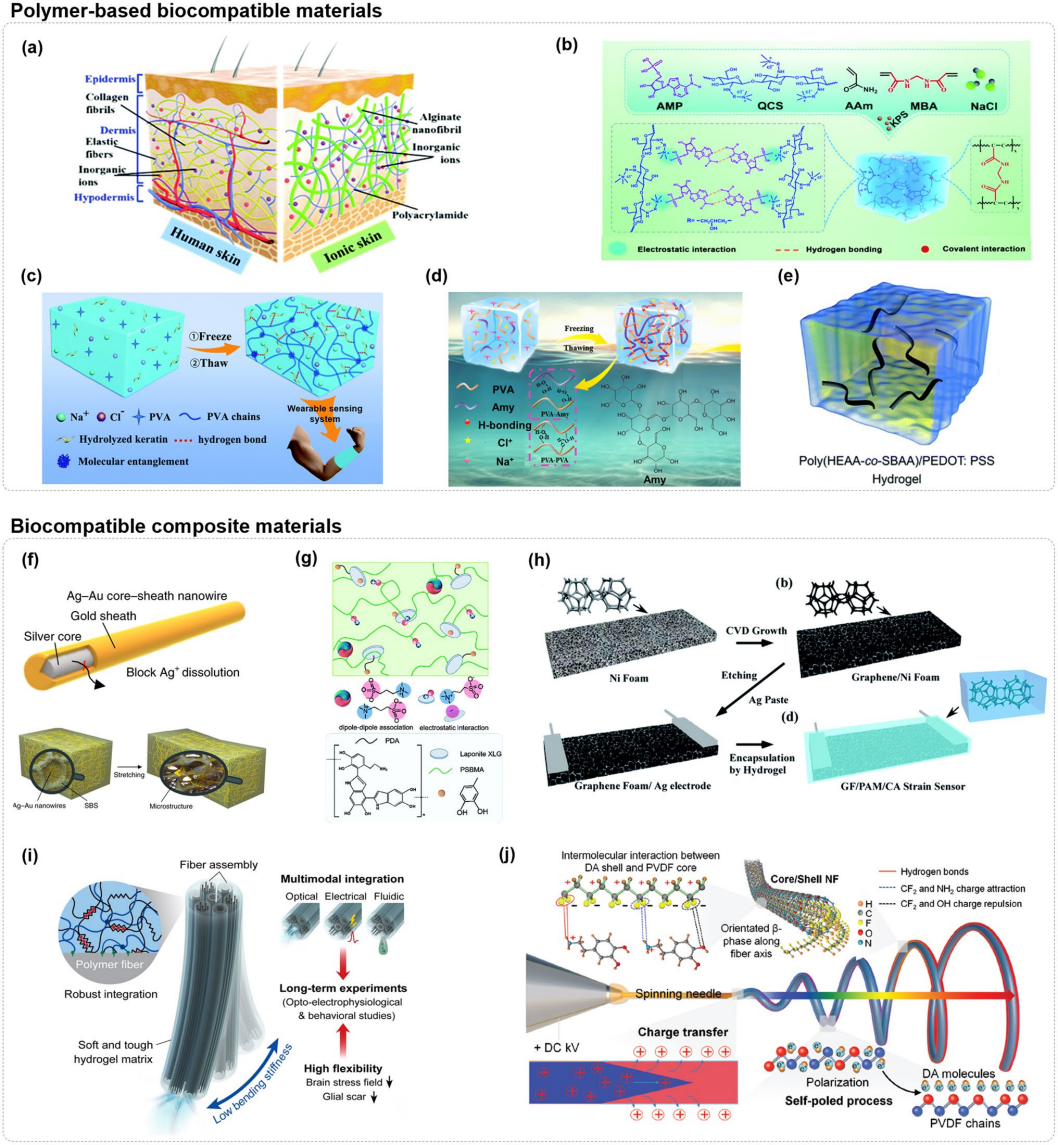
Recent progress of the biocompatible materials for implantable devices: Polymer-based materials (**a**–**e**), composite materials (**f**–**j**). **a** Reproduced with permission [157] Copyright 2019, RSC. **b** Reproduced with permission [158] Copyright 2021, RSC. **c** Reproduced with permission [159] Copyright 2020, RSC. **d** Reproduced with permission [160] Copyright 2022, RSC. **e** Reproduced with permission [161] Copyright 2020, RSC. **f** Reproduced with permission [164] Copyright 2018, Springer Nature. **g** Reproduced with permission [14] Copyright 2020, RSC. **h** Reproduced with permission [165] Copyright 2019, RSC. **i** Reproduced with permission [166] Copyright 2021, Springer Nature. **j** Reproduced with permission [167] Copyright 2021, Springer Nature

Along with the tunable nature of polymers, the elasticity of polymers has led the development of biocompatible polymers that are mechanically matched to the desired implantation site. This allows the use of polymers as biocompatible substrate materials and also as conductive sensing materials [184, 185]. Many of the biocompatible polymers discussed in this section were used as on-skin strain and pressure sensors, but are sufficiently biocompatible for implantation. An ionic supramolecular nanofibrillar double-network (NaCl/SA/PAM) hydrogel was developed by Zhang et al. [157] (Fig. 7a). The NaCl/SA/PAM hydrogel aims to replicate the mechanical and sensing capabilities of the human dermis by using network structures of cross-linked supramolecular sodium alginate (SA) nanofibrils, cross-linked polyacrylamide (PAM), and salting-out with NaCl to mimic supramolecular collagen fibers, elastin 3D networks, and other inorganic ions, respectively. The novel NaCl/SA/PAM hydrogel can be injected into complex shapes, and reach strains of up to 3120% without fracture, with a skin-mimicking elastic modulus of < 70 kPa. The hydrogel also displayed excellent strain-sensing capabilities for a broad range of 0.3–1800% strain.

Rather than mimicking skin, Zhang et al. [158] also used a different approach, developing a biocompatible, DNA-inspired hydrogel (QCS-AMP/PAAm) mechanoreceptor with skin-like mechanical properties and excellent biocompatibility (Fig. 7b). The hydrogel was designed using adenosine monophosphate (AMP) as the nucleotide model, while quaternized chitosan (QCS) was selected to mimic DNA chains. This DNA-mimetic network structure was then incorporated into a covalently cross-linked polyacrylamide (PAAm) network. The resulting hydrogel displayed excellent mechanical properties, with a fracture strain of 1731%, high toughness of 1457.6 kJ m^−3^, and an elastic modulus of 22.9 kPa. With the incorporation of NaCl, the hydrogel displayed good conductivity with a gauge factor of about 3.38. Chen et al. [162] developed an alginate hydrogel iontronic fiber (AHIF), designed to mimic both the mechanical and electrical properties of soft biological tissue. A simplified wet-spinning process was used to spin the AHIF. Ca^2+^ ions were chelated with alginate molecules to form a cross-linking network of alginate. This network then underwent gelation and was spun to create the AHIFs. The conductivity of the AHIFs ranged between 0.054–0.105 S m^−1^, matching the electrical properties of biological tissues such as the heart (0.054 S m^−1^), lungs (0.039–0.203 S m^−1^) and muscle (0.202 S m^−1^). The AHIFs could be structurally rearranged through mechanical training to form highly oriented molecular networks with stress–strain curves and self-stiffening properties similar to biological tissue. Gao et al. used a simpler approach to synthesizing another hydrogel mechanoreceptor, with a focus on biocompatibility and eco-friendliness [159] (Fig. 7c). The hydrogel was fabricated in a one-step freeze–thaw method, in which hydrolyzed keratin (HK), NaCl and PVA were mixed, frozen, and then thawed to form the HK/PVA/NaCl hydrogel. By introducing hydrolyzed keratin and NaCl into the PVA hydrogel, a reversible physically cross-linked network was created between PVA and HK, enabling skin-like mechanical properties and recyclability. Salting-out with NaCl induced entanglement of the PVA chains, further contributing to the hydrogel’s mechanical strength. anti-fatigue features, and ionic conductivity. Another hydrogel was developed by Gao et al. [160] focusing on increased anti-swelling properties on top of skin-like mechanical properties, simple synthesis process, and biocompatibility (Fig. 7d). To minimize use of environment-unfriendly chemical cross-linkers or organic solvents to reduce swelling, Gao et al. used a similar one-step freeze–thaw method to introduce hyperbranched amylopectin (Amy) into polyvinyl alcohol (PVA), with NaCl salting-out to form a PVA/Amy/NaCl hydrogel that displayed excellent skin-like mechanical properties and strain sensitivity. The previous hydrogels discussed achieved conductivity through incorporation of NaCl salt ions. However, fillers can be incompatible with their polymeric matrix, compromising the hydrogel’s stretchability, recyclability, and robustness. As a result, Zhang et al. [161] have developed conductive polymers without the use of fillers, incorporating PEDOT:PSS into a zwitterionic poly(HEAA-*co*-SBAA) network to form poly(HEAA-*co*-SBAA)/PEDOT:PSS hydrogels that are stretchable, self-adhesive, biocompatible, and strain-sensitive (Fig. 7e). The resulting hydrogel displayed excellent mechanical properties, with up to 5000% stretchability. The poly(HEAA-*co*-SBAA)/PEDOT:PSS also demonstrated a high conductivity of 0.625 S m^−1^ at low strain, and a gauge factor of up to ~ 11. Zhang et al. [163] focused on the creation of structurally and mechanically tissue-matching biomaterials through the development of a biomimetic meso-assembly processing engineering (MAPE) process based on animal silks. Reconstituted silkworm silk fibroin (RSSF) was combined with polyethylene oxide (PEO) and Ca^2+^ ions to form a biomimetic meso-assembling film (BMAF). Mechanical training was used to structurally remodel the RSSF globules into nanofibrils with highly oriented cross-linked molecular networks within. The resultant-oriented BMAFs (O-BMAFs) demonstrated good balance between strength (5 ± 1 MPa), stiffness (18 ± 2 MPa), and toughness (6 ± 1 MJ m^−3^), with extensibility and moduli comparable to that of skin, muscles, and eye lenses.

#### Composite Materials

When a device requires multiple properties difficult to achieve with a single homogenous material, composite materials are often used to combine the benefits of multiple different materials [139, 141]. Metals and carbons are often used as fillers to imbue conductivity and increased sensing capabilities. Biocompatible polymers are often used as substrates to impart stretchability and stability to sensing materials, or to encapsulate non-biocompatible materials to render the resulting sensor biocompatible. By combining materials, resulting composites are increasingly versatile and can encompass a wide range of functions and applications. Choi et al. developed an Ag–Au core–sheath nanowire composite for continuous recording of myocardial electrophysiology and cardiac tissue stimulation using an implantable cardiac mesh [164] (Fig. 7f). Silver nanowire is a popular filler in conductive nanocomposites due to its high conductivity and easy mass production; however, leaching of silver ions into the body can cause adverse health effects. By coating a silver nanowire core in a gold sheath before incorporation in a polymer matrix, the biocompatibility of the nanowires is improved while maintaining the high conductivity of silver nanowire. Using the Ag–Au core–sheath nanowire percolation network as a filler in the poly(styrene–butadiene–styrene) (SBS) elastomer matrix, a nanocomposite was developed with both high stretchability and conductivity, with an optimized stretchability of 266% with a Young’s modulus of 15 MPa, and an optimized conductivity of 41,850 S cm^−1^. Fillers were also used by Pei et al. [14] in a zwitterionic nanocomposite hydrogel focused on robust adhesion to dry and wet surfaces, for use as strain sensors in organ motion sensors (Fig. 7g). The hydrogels were synthesized using a simple one-pot free radical polymerization process combining sulfobetaine methacrylate (SMBA) monomers with dopamine-modified clay and MBAA cross-linking agent. The presence of both zwitterionic monomers and catechol groups impart robust hydrogel–tissue adhesion; zwitterionic monomers within the hydrogel provide dipole–dipole interactions, while free catechol dopamine groups permit physical and chemical bonding at the hydrogel–tissue interface. As a result, the conductive hydrogels can reach over 1000% strain and adhesion strength of 19.4 kPa on biotissues, with a gauge factor of 4.3 for a strain range of 0–670%. Polymers can also provide scaffolding and support for other materials, creating different forms of composites. This approach was taken by Cai et al. [165], who encased graphene foam in a hydrogel to create a novel, on-skin graphene strain sensor (GF/PAM/CA) for motion detection (Fig. 7h). While graphene has faced difficulties in microscale patterning, by using a 3D foam, a simplified process allowed for bulk synthesis while preserving graphene’s conductivity and mechanical properties. To form the graphene foam, graphene was chemical vapor deposited upon a template, which was then removed with an etching solution. A double-network PAM/CA hydrogel was then infiltrated into the graphene foam, lending increased stretchability and stability. Chitosan was chosen to imbue adhesive properties to the GF/PAM/CA composite; its ability to form strong bonds with the tissue surface allows strong adhesion to the implantation site. Using this hydrogel, a large range of strains can be measured up to 500% with a gauge factor of ~ 1800, and adhesion energy of ~ 200 J m^−2^. Encapsulation was similarly used by Park et al., who used a hydrogel-encased functional fiber hybrid probe that interfaces with brain tissue for extended periods of time [166] (Fig. 7i). This neural interface comprised of bundled functional fibers in a PAAm-Alg hydrogel matrix. Three functional fibers were used: polymer optical and microfluidic fibers, and a composite of tin and polymer for microelectrode arrays. Using a bundled-fiber approach, a substantially lower bending stiffness as achieved compared to conventional probes; the surrounding hydrogel serves to mechanically isolate each fiber, creating distinct stress fields and reducing bending stiffness. The bending stiffness of the hydrogel hybrid probes depends on their hydration state, allowing them to mechanically adapt and decrease in bending stiffness post-implantation, with fully swollen hybrid probes having a bending stiffnesses measuring 7 N m^−1^. Piezoelectric materials are increasingly of interest in biomaterial development due to their broad applicability toward implants such as energy harvesters, tissue stimulators, mechanical sensors, and more [168, 186]. However, many conventional piezoelectric materials such as lead zirconium titanate (PZT) are lead-based ceramics, resulting in brittleness and toxicity concerns that make them undesirable as implantable biomaterials [187]. As a result, biocompatible piezoelectric materials have been developed for use in healthcare applications. Piezoelectric polymers such as PLLA have been especially of interest due to their inherent stretchability and flexibility and their highly tunable nature. However, many piezoelectric polymers demonstrate low piezoelectric coefficients. As a result, piezoelectric composites have been developed to combine the advantages of piezoelectric polymers with the high piezoelectric coefficients of inorganic piezoelectric materials [188], further bypassing the inherent brittleness common to many inorganic piezoelectric materials [186, 189]. Li et al. [168] developed a piezoelectric composite nanofiber (NF), creating an all-fiber soft sensor able to detect weak physiological mechanical stimulation such as blood pulsation. In order to create this nanofiber sensor, poly(vinylidene fluoride) (PVDF) was chosen for its flexibility and biocompatibility. In order to combat its weak piezoelectric effect and low ferroelectric stability, dopamine was introduced to the PVDF solution, which was then electrospun. Through high stretching forces present during electrospinning, the PVDF is converted into its more electrically active β-phase [190], while simultaneously orienting the nanofibers. Dopamine was used as a nanofiller to promote nucleation of β-phase PVDF and maintain the stability of β-phase PVDF once oriented. Flexible thin films were created through random stacking of these PVDF/DA nanofibers. The resulting piezoelectric motion sensors were demonstrated to have great sensitivity and accuracy in measuring diaphragmatic and arterial movements when tested in vivo in mice.

### Bioresorbable Materials

Most implanted sensors require the biostability of the sensor, so that the device does not degrade and cease to function over time [135]. However, an implanted sensor with a limited lifetime can be desirable; with a resorbable implant, further surgical intervention to remove the implant at the end of its functional lifetime is not necessary [191]. However, additional constraints are demanded of bioresorbable materials. In a bioresorbable implant, all materials and their degradation products must be biocompatible [192]. The materials must also degrade only after the expected lifespan of the sensor has elapsed [21, 192]. In this section, recent developments regarding bioresorbable materials are discussed, with special focus on bioresorbable metals, silicon-based materials, polymers, and composite materials. Table 3 shows comprehensive information about various type of bioresorbable materials for implantable electronics by comparing core features including cytotoxicity, dissolution rate, mechanical modulus, and so on.

**Table 3:** Various type of bioresorbable materials for implantable electronics

Type	Material	Cytotoxicity	Dissolution rate	Mechanical property		Electrical property	Application	Refs
				Stretchability (%)	Modulus (MPa)	Resistivity		
Metal	Double-layered W/Mg	No significant changes in the myocardial wall 3 weeks after implantation	W: 96 nm day^−1^ Mg: 7.2 um day^−1^	< 0.6	45,000 (Mg)	4e^−6^ Ω cm	Electrode for cardiac pacemaker	[193]
	Mg, Mo thin films	No signs of inflammation at 8 weeks post-implantation	9 h electrode lifetime	–	–	Near zero	Nerve stimulation device electrodes	[194]
	Polycrystalline Mo	–	8 nm day^−1^	–	–	–	Spectroscopic biomarker electrodes	[195]
	MgZnCa metallic glass	97% cell viability after 48 h	3500 nm h^−1^	≈ 115	49,000	8.55e^−5^ Ω cm	Mechanical energy harvesting	[196]
	Iron–manganese alloy	95% cell viability	~ 34 days (1.5 um film)	–	–	–	Nerve regeneration device electrodes	[197]
Silicon	Phosphorus-doped Si NM	No moderate astrogliosis 4 weeks post-implant	1 month	–	–	–	Spatiotemporal cerebral cortex mapping electrode array	[198]
	Si NM, SiO2, nanoporous Si thin layer	Inflammatory cell infiltration and fibrosis comparable to control	23 nm day^−1^ (Si NM)	–	–	249 kΩ	Strain gauge, SiO2 biofluid barrier, substrate for intercranial pressure sensor	[199]
	Thermally grown SiO2 on Si wafers	Absence of abnormalities in the major organs	0.11 nm day^−1^ (t-SiO_2_);	–	–	–	Intercranial pressure sensor	[200]
	Si NM diaphragms	Absence of abnormalities after 5 weeks in major organs	Estimated ~ 195 days	–	–	7–8 kΩ	Optical intercranial pressure, temperature sensors	[201]
Polymer	Bioresorbable dynamic covalent polyurethane (b-DCPU)	No substantial skin necrosis or swelling after 4wks	~ 4.78 × 10–5 g day^−1^	170–340	0.51–3.76	–	Substrate and biofluid barrier	[202]
	PLLA/PCL	–	~ 100 days	–	10	476 Ω	PLLA/PCL substrate and encapsulation layer for ECoG device	[203]
	BTIM	95.6% cell viability (cured BTIM)	Several months (PEG-2LA-DA system)	Up to tenfold	0.03	2e^10^ Ω cm	Adhesive, encapsulation of electrophysiology mapping device	[204]
	PBTPA	No substantial skin necrosis or swelling after 4 wks	Degradation rates of 0.05–1.15 mg day^−1^	27.5–32.7	5.2–25.1	–	Encapsulation layer and biofluid barrier	[202]
Composite	Br-doped Si NM electrodes coated in FeNPs	–	≈15 h	–	–	–	Electrodes for dopamine biomolecule sensor	[205]
	Candelilla wax/WNPs paste	No significant changes in myocardial wall in 3 weeks	–	–	–	–	Fully implantable cardiac pacemaker	[206]
	CSFH	–	–	Up to 100	≈ 0.001–0.15	–	Intracranial pressure sensing electrodes	[207]

#### Metal-Based Materials

**Fig. 8:**
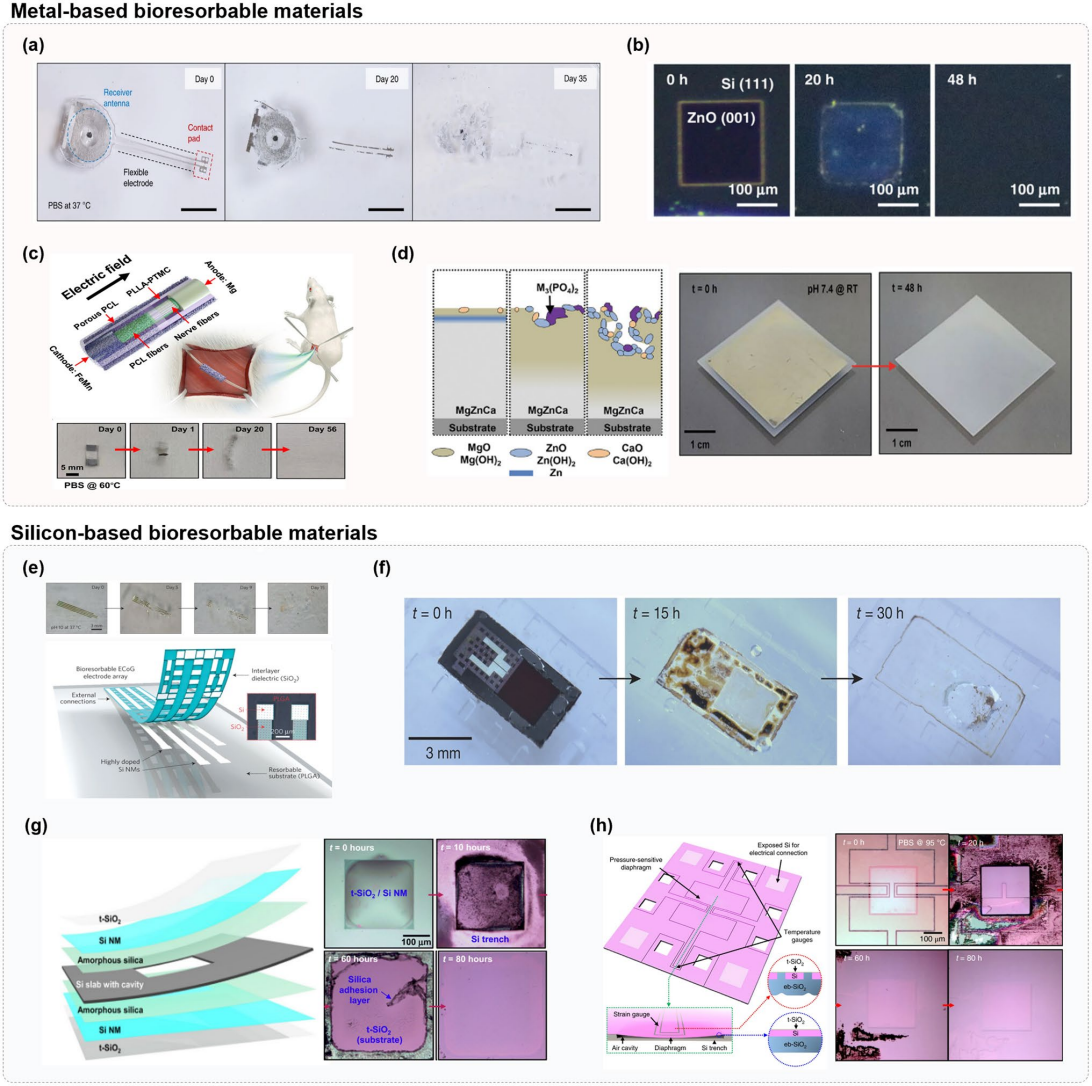
Recent progress of the bioresorbable materials for implantable devices: Metal-based materials (**a**–**d**), silicon-based materials (**e**–**h**). **a** Reproduced with permission [194] Copyright 2021, Springer Nature. **b** Reproduced with permission [195] Copyright 2021, Wiley-VCH. **c** Reproduced with permission [197] Copyright 2020, AAAS. **d** Reproduced with permission [196] Copyright 2019, Wiley-VCH. **e** Reproduced with permission [198] Copyright 2016, Springer Nature. **f** Reproduced with permission [199] Copyright 2016, Springer Nature. **g** Reproduced with permission [200] Copyright 2019, AAAS. **h** Reproduced with permission [201] Copyright 2018, Springer Nature

Most metals are not bioresorbable; in aqueous environments, they corrode and oxidize, often creating toxic products [208–213]. However, the resorbable metals for transient implantable sensors have proved attractive, due to metal’s high conductivity and ease of fabrication [192, 214]. Magnesium (Mg), zinc (Zn), and molybdenum (Mo) are three of the most used bioresorbable metals due to their high biocompatibility [218]. Due to their differing degradation pathways and dissolution kinetics, different metals may be selected based on expected functional lifetime, any necessary encapsulation and processing [21, 191]. Metal thin films were used to create fully resorbable implantable electronics; Koo et al. [194] using magnesium (Mg) and molybdenum (Mo) in a resorbable neurogenerative therapy device (Fig. 8a), Choi et al. [193] used magnesium and tungsten (W) in resorbable cardiac pacemakers, and Lu et al. [195] used Mo and W to develop LEDs for photodynamic therapies (Fig. 8b). While the studies used metal thin films as conductors, the metals chosen differed in their rate of resorption; magnesium has the fastest resorption rate of 7 × 10^–2^ μm h^–1^ at room temperature in representative biofluid, tungsten degrades at a rate of (1.7–0.3) × 10^–3^ μm h^–1^, and molybdenum has the slowest resorption rate at 3 × 10^–4^ μm h^–1^ [215]. Rather than just using a monometallic thin film, Wang et al. took a different approach, developing electrodes using an alloy of iron and manganese (FeMn) for a microscale conduit device promoting peripheral nerve regeneration [197] (Fig. 8c). To sustain electric fields for the nerve regeneration device, Mg and FeMn electrodes were used as the anode and cathode of a galvanic cell, respectively, with biofluids as the electrolyte. FeMn alloy was chosen as Fe and Mn have been used for biocompatible stents; the alloy also had a suitable electrochemical potential for the galvanic cell, a corrosion rate three times faster than pure Fe, and antiferromagnetic character, making FeMn compatible for use with an MRI and magnetron sputtering techniques. Unlike previous sensors that used more traditional forms of metal, Bae et al. used a different approach using a metallic glass (MG) made using magnesium (Mg), zinc (Zn), and calcium (Ca), with which implantable triboelectric nanogenerator and temperature sensors were demonstrated [196] (Fig. 8d). Due to their amorphous nature and lack of crystal defects, MGs can reach much higher strains compared to traditional metals and maintain their electrochemical properties even in aqueous solution, and nanofilm fabrication can easily be performed using established method. The fabricated nanoscale MgZnCa metallic glass had an elastic modulus of 49.0 GPa and fracture strain of 2.9%, compared to the elastic modulus and fracture strain of Mg at 36.8 GPa and 2.2% strain, respectively. Serpentine MgZnCa electrodes on PBAT were able to reach much higher strains; resistance was stable up to 115% strain and doubled at 140% strain, while similarly fabricated Mg electrodes were electrically stable only up to 75% strain.

#### Silicon-Based Materials

With a well-established history of usage in the electronics industry due to its excellent semiconducting properties, silicon remains important in the realm of biomedicine. Bulk silicon (Si) is inelastic and non-resorbable, but when the dimensions of silicon are reduced to the nanoscale [142, 192, 216], silicon nanomembranes are bendable and deformable [142, 216, 217], and resorb easily into biofluids. While nanoscale silicon has demonstrated higher levels of toxicity compared to bulk silicon, its in vivo toxicity remains acceptably low, being cleared quickly from the body after degradation. The use of Si NM is demonstrated in Kang et al., Shin et al., and Yu et al.; both Kang and Shin used Si NM in intracranial pressure and temperature sensors [199, 200], while Yu used Si NMs to develop an ECoG electrode array for spatiotemporal cerebral cortex mapping [198] (Fig. 8e). The Kang and Shin intracranial pressure sensors use both single-crystalline Si NMs and thin wafers of silicon to form biodegradable diaphragms. Si NMs are used for their high optical transmittance, flexibility, and quick dissolution rates, while silicon wafer substrates have slower dissolution rates, but provide more stability and are used as substrate [199] (Fig. 8f). In contrast, the temperature sensors by Shin et al. use Si NMs that have been patterned with nanoscale arrays of holes; the holes create a photonic crystal (PC) structure that allow changes in temperature and pressure to be measured [200] (Fig. 8g). In all three sensors, the Si NM is highly doped with phosphorus to form *n*-doped Si NMs, allowing the NMs to be used as electrodes by themselves. Doping with phosphorus changes the dissolution rates, however, slowing it down to ∼11 nm d^−1^. Unlike Si NMs, silicon oxides are more often used as dielectric layers, or as biofluid barriers. While Si NM are typically chosen for their quick resorption times, encapsulation is necessary to protect devices throughout their expected functional lifetime. SiO_2_ has several benefits over polymers for encapsulation [201] (Fig. 8h). Hydrophilic polymers swell in water, causing fracturing of the polymer, water permeation, and loss of function before the encapsulating layer is fully resorbed. Shin et al. [201] uses thermally grown SiO_2_ as a biofluid barrier as they are defect-free over large areas, letting it resorb evenly at slow rates of several hundredths of a nanometer per day, protecting devices from water permeation for weeks at a time.

#### Polymer-Based Materials

**Fig. 9:**
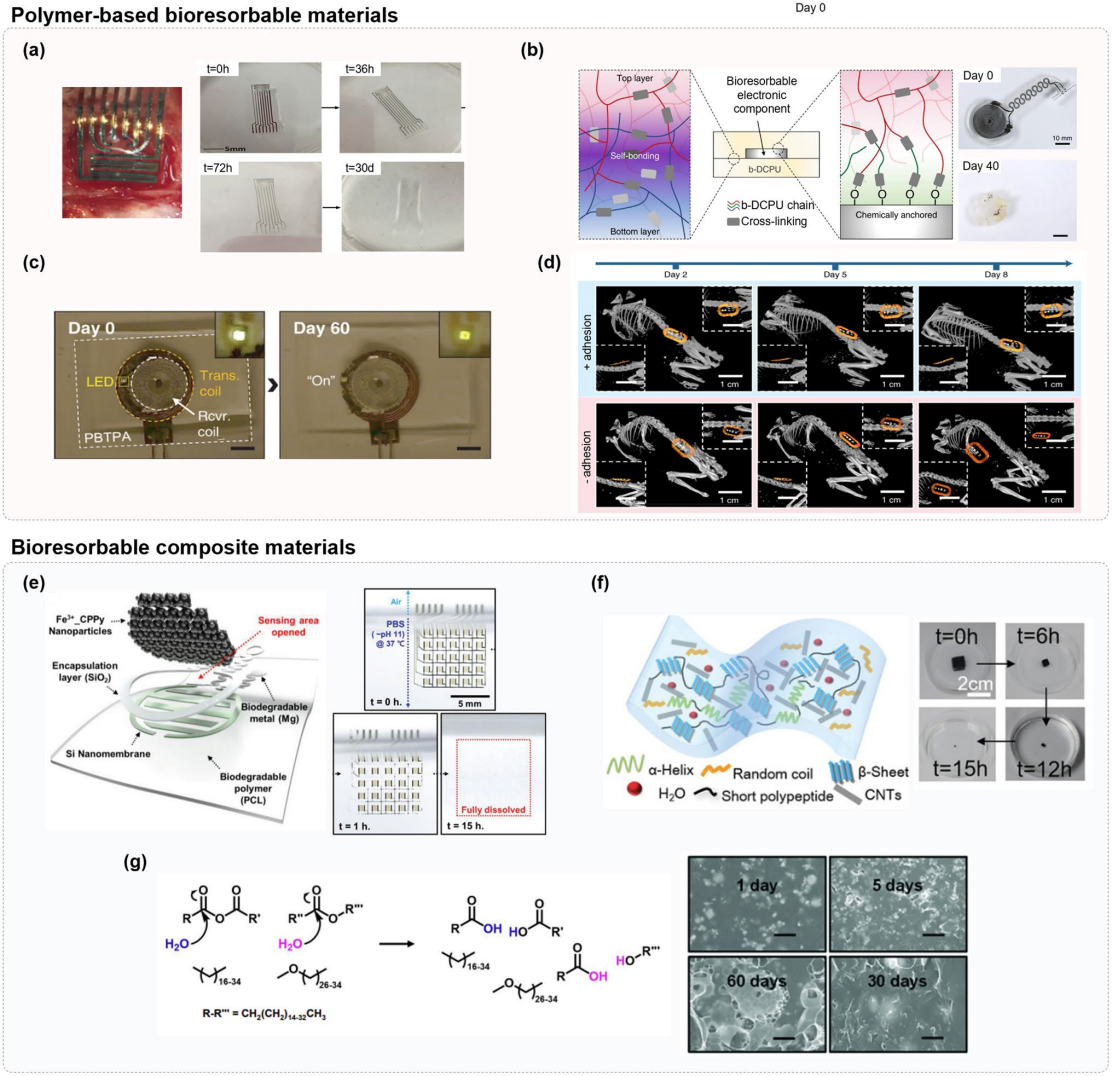
Recent progress of the bioresorbable materials for implantable devices: Polymer-based materials (**a**–**d**), composite materials (**e**–**g**). **a** Reproduced with permission [203] Copyright 2019, Wiley-VCH. **b** Reproduced with permission [222] Copyright 2020, Springer Nature. **c** Reproduced with permission [202] Copyright 2020, Wiley-VCH. **d** Reproduced with permission [204] Copyright 2021, Springer Nature. **e** Reproduced with permission [205] Copyright 2018, Wiley-VCH. **f** Reproduced with permission [207] Copyright 2020, Wiley-VCH. **g** Reproduced with permission [206] Copyright 2018, Wiley-VCH

The versatility of polymeric materials has long made them an attractive option for resorbable electronics. By adjusting the properties of polymers during synthesis, such as molecular weight, crystallinity, or hydrophilicity, the dissolution rate of polymers can easily be tuned [192, 218, 219]. As a result, bioresorbable polymers are often used as substrates, or encapsulation materials and biofluid barriers for bioresorbable sensors [139, 192, 214, 219–221]. Xu et al. [203] demonstrates a novel PLLA/PCL composite for use as a resorbable substrate and encapsulation material for a bioresorbable electrocorticography (ECoG) electrode array and intracranial pressure (ICP) sensor (Fig. 9a). The PLLA/PCL (80:20) composite was chosen as a substrate and encapsulation material for its long degradation time and good malleability. The electrodes encapsulated in 10 µm PLLA/PCL worked stably for 5 days, with PLLA/PCL taking about 100 days to dissolve in PBS completely. PLLA/PCL had an elastic modulus of ~ 10 MPa and elongation rate of 30%, suggesting good conformity with biotissue. A different approach was taken by Choi et al. [222], using the elastomeric properties and decreased swelling in aqueous solution of a novel bioresorbable dynamic covalent polyurethane (b-DCPU) in soft, long-lived bioresorbable electronic stimulators (Fig. 9b). b-DCPU contains dynamic covalent bonds, allowing the reversible rearrangement of bonds; thermally activated dynamic bond exchange reactions imbue strong self-adhering properties, while oxygen plasma treatment allows bonding between b-DCPU and other inorganic sensor materials, leading to long-term electrical stability. With a variable cross-linking density, b-DCPU acts as an effective biofluid barrier; full degradation of 200 µm b-DCPU takes over a year in PBS.

While most bioresorbable polymers used as biofluid barriers remain hydrophilic, displaying swelling behavior in biofluids, a novel polyanhydride-based polymer (PBTPA) was developed by Choi et al. [202] that displayed a hydrophobic character instead (Fig. 9c). Rather than through swelling and dissolution, degradation occurs through surface erosion with little swelling, allowing PBTA to act as an effective and transient encapsulation layer. Using different ratios of precursor, the cross-linking ratio of PBTPA could be modified, changing its properties. Using 50 µm PBTPA as an encapsulating material, a non-resorbable LED was immersed in PBS; the LED’s emitted intensity remained stable for 50 days before fully failing after 70 days, showcasing PBTPA’s effectivity as a fluid barrier. Yang et al. [204] also went in an alternative direction from polymer substrates and encapsulants, developing a novel bioelectronic–tissue interface material (BTIM) to adhere implanted bioelectronics and soft biological tissue together (Fig. 9d). The BTIM uses a combination of photocurable PEG-LA-DA covalent networks and sodium alginate ionic networks that form quickly after application to the electronic–tissue interface. These ionic networks facilitate an ionic conductivity of ~ 0.5 S m^−1^, allowing for use in a variety of optoelectronic and bioelectronic devices. After curing, the BTIM retains good mechanical properties; a Young’s modulus of ~ 30 kPa and an up to tenfold elongation minimizes stress and damage at the tissue–device interface, while the BTIM has a high cohesion energy of 1900 J m^−2^. Resorption of the adhesive takes place through hydrolysis of lactide repeat units; by modifying the lactide content of PEG-LA-DA, the degradation speed of BTIM ranges from ~ 20 days to several months in PBS.

#### Composite Materials

Like biocompatible composite materials, bioresorbable composites combine different material types into a single material; however, they differ in that all constituent materials must again be bioresorbable. By coating silicon nanomembranes in catalytic iron (Fe) nanoparticles, Kim et al. [205] developed flexible, transient electrochemical dopamine monitors (Fig. 9e). Fe was used as an alternative to commonly used but non-transient platinum (Pt) nanoparticles due to their similar catalytic performance in response to dopamine, and ability to selectively measure physiological concentrations of dopamine among other neurotransmitters. While there were negligible changes in current when solution of neurotransmitters without dopamine was flowed over the dopamine sensor, an appreciable change in current was detected with solution containing 1 × 10^–6^ M of DA. The sensor was also able to fully dissolve in PBS solution at physiological temperatures, with most components dissolving fully in 15 h. The doping of a substrate to form nanocomposites was also performed by Zhang et al. [207], who developed a conductive silk hydrogel (CSFH) composite for use as an implantable ICP sensor (Fig. 9f). The mild conditions necessary for silk processing make it easy to modify silk and add functional dopants to suit the desired application; in the case of this composite, carbon nanotubes were used to dope the silk hydrogel to imbue greater conductivity to the material while maintaining the desired mechanical properties. The CSFH displayed good mechanical properties, with an elastic modulus of between 0.001 and 0.15 MPa and was able to reach 100% strain without a significant decrease to its sensing properties or permanent deformation. Won et al. [206] took a similar approach to develop bioresorbable materials for interconnects and encapsulants, using natural waxes for their hydrophobic and biodegradable nature (Fig. 9g). Candelilla wax was found to have the greatest hydrophobicity performance of the three natural waxes assessed, displaying almost no water uptake after 15 days of immersion when used to encapsulate devices. Through the addition of tungsten nanoparticles, a conductive C-wax was developed, with conductivity of up to 4 kS m^−1^. However, the mechanical properties of waxes may be lacking for some applications; while waxes in layers smaller than 200 µm can bend up to a radius of 20 mm, cracks begin to appear at thicknesses of 300 µm at the same bending radius.

## Applications

### Cardiovascular Monitoring

**Fig. 10:**
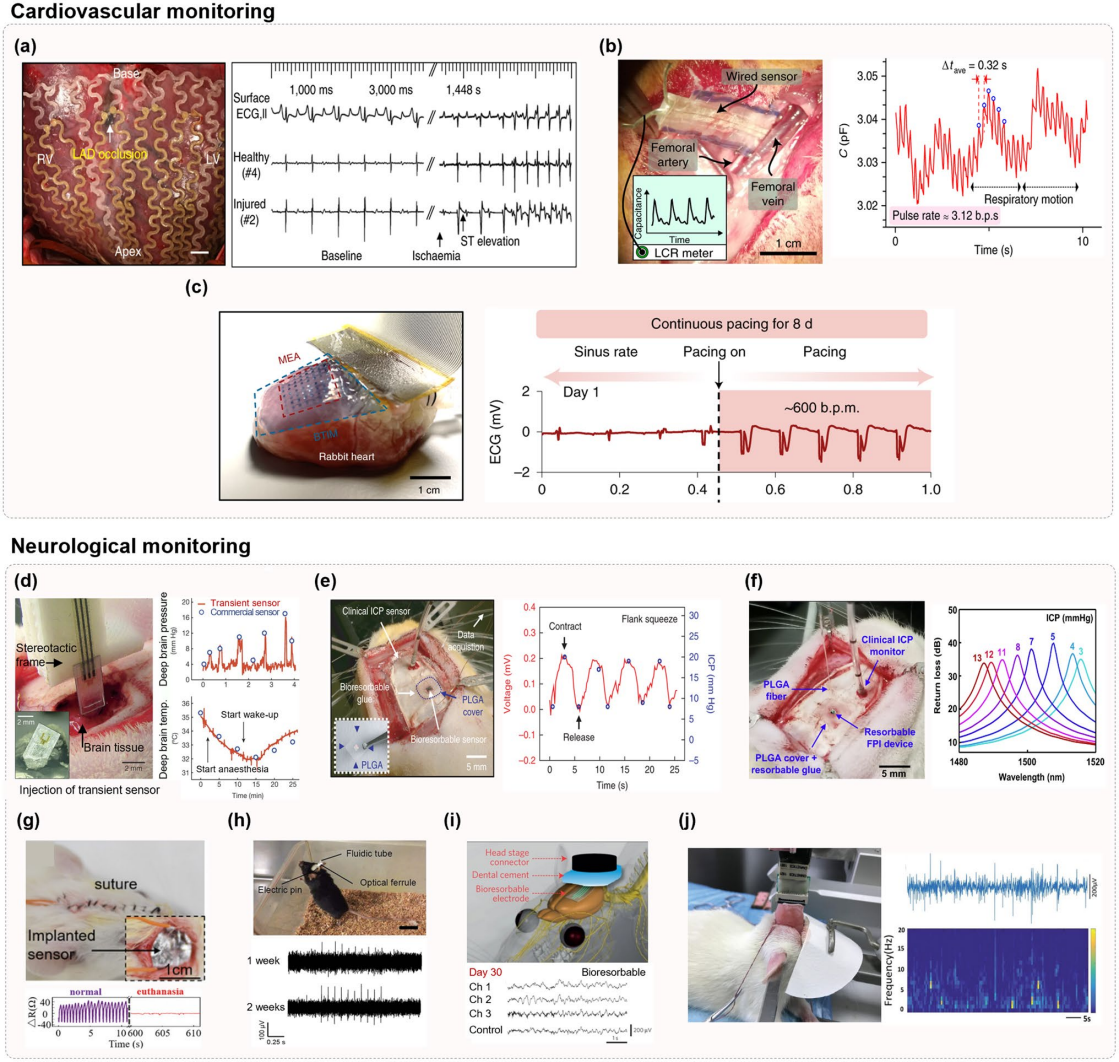
Advanced wireless, batteryless, implantable electronics system for in vivo physiological signal monitoring applications: Cardiovascular monitoring (**a**–**c**), neurological monitoring (**d**–**j**). **a** Reproduced with permission [164] Copyright 2018, Springer Nature. **b** Reproduced with permission [223] Copyright 2019, Springer Nature. **c** Reproduced with permission [204] Copyright 2021, Springer Nature. **d** Reproduced with permission [199] Copyright 2016, Springer Nature. **e** Reproduced with permission [201] Copyright 2018, Springer Nature. **f** Reproduced with permission [200] Copyright 2019, AAAS. **g** Reproduced with permission [207] Copyright 2020, Wiley-VCH. **h** Reproduced with permission [166] Copyright 2021, Springer Nature. **i** Reproduced with permission [198] Copyright 2016, Springer Nature. **j** Reproduced with permission [203] Copyright 2019, Wiley-VCH

Researcher's prior integration of bioelectronic systems into cardiovascular applications brings a distinct set of breakthroughs and challenges. Choi et al. [164] developed an Ag–Au nanocomposite to create electrodes to measure cardiac electrophysiological signals (Fig. 10a). While soft cardiac devices have previously been demonstrated in a rat model, prior to the work by Choi et al., no work had been done on larger models such as a swine model, that reflect human physiology more accurately. A cardiac mesh was designed based on a simplified version of the swine heart, using seven repetitive segments of six electrodes welded together to form a 2D fan shape. Using this fan shape, the cardiac mesh can completely cover the swine heart’s ventricles when folded, allowing the mesh to sense and stimulate multiple locations on the swine heart with specificity. In addition to electrophysiological signals, sensors measuring other cardiovascular phenomena have been developed. A biodegradable, wireless and battery-free arterial blood flow sensor was developed for continuous monitoring post-discharge and to remove the need for further surgical intervention to remove the sensor [223] (Fig. 10b). A poly(glycerol sebacate) (PGS) dielectric layer was formed with pyramidal microstructures for increased sensitivity in pressure-sensitive regions. Poly(octamethylene maleate (anhydride) citrate) (POMaC) and polyhydroxybutyrate/polyhydroxyvalerate (PHB/PHV) were used as packaging layers, with stiffer PHB/PHV in contact with muscle and softer POMaC in contact with the artery to create a cuff more sensitive to arterial deformation than respiratory movements. Two narrow variable capacitors are also used instead of a single larger one to increase the flexibility of the sensor, to allow wrapping of the cuff around arteries with diameters less than 1 mm.

Like the previously discussed sensors, the integration of bioelectronics often requires the use of sutures that can result in insufficient conformal contact or tissue damage. Existing tissue adhesives can serve as alternatives; however, they often lack sufficient adhesion to wet tissues and in electrical conductivity. A novel BTIM adhesive was used instead to encapsulate and adhere an interconnected array of 64 electrodes to rabbit hearts for use in electrical spatiotemporal mapping by Yang et al. [204] (Fig. 10c). In a constant-pressure system, the BTIM-adhered electrode system was able to measure high-quality normalized electrograms, with amplitudes comparable to previously developed devices. Adhesion was also found to remain stable after eight hours with minimal delamination. By using a novel BTIM adhesive, Yang et al. adhered sensors for cardiac electrophysiology mapping with greater stability, conformation, and adhesion strength than previous devices.

### Neurological Monitoring

Previous research on bioelectronic system for neurological monitoring shows unique developments and challenges. a silicon nanomembrane-based ICP strain sensor was developed by Kang et al. [199] that can resorb completely within a span of days in aqueous solution (Fig. 10d). The sensor consisted of a diaphragm with serpentine silicon nanomembrane (Si NM) as the piezoresistive element. The sensor platform was easily adapted through modification for fluid flow, motion, pH, or thermal property sensing, allowing the sensor to adapt to many clinical needs. Unlike other devices described in this paper, the device demonstrated by Kang et al. was connected to an external wireless communicator through Mo percutaneous wires, which can lead to secondary infection at the wires. However, the use of the sensor with an implanted wireless—though only partially resorbable—communicator using near-field communication technology was demonstrated, showing that a fully wireless sensor is achievable. Though previously developed ICP sensors have demonstrated comparable sensitivity and accuracy to existing, conventional sensors, they often lack operational lifetimes adequate for many applications. As such, Shin et al. [201] focused on developing a resorbable ICP sensor with an extended lifetime spanning weeks (Fig. 10e). The ICP sensor featured four silicon nanomembrane sensors; two strain sensors built upon an air-filled flexible diaphragm, and two temperature sensors. The device was then encapsulated using t-SiO_2_ thin films as a biofluid barrier. Thermally grown layers of silicon dioxide (t-SiO_2_) degrade at slow rates of several hundredths of a nanometer per day. Failure of t-SiO_2_ as a biofluid barrier largely occurred due to hydrolysis rather than water permeation through defects or the material itself. When tested in vivo, the t-SiO_2_ ICP sensor experienced little drift during the first 18 days of implantation. After 25 days of implantation, the drift experienced was comparable to that of conventional ICP monitors several days after implantation without recalibration. As a result, the use of t-SiO_2_ as a biofluid layer enabled long-term monitoring of intracranial pressure in rats for over 25 days.

The same group developed a second sensor using similar diaphragms to measure intracranial pressure. However, rather than measuring the strains experienced by the diaphragm, a combination of mechanical and optical changes in properties of photonic crystal (PC) structures were used [200] (Fig. 10f). Nanoscale holes, forming PC structures, were created on the sensor's diaphragm. As pressure-induced deflections cause changes in the PC lattice parameters, it causes a shift in resonant peak positions. Through use of a free space detection system, the optical sensor can operate wirelessly without tethering to an external source/detector system. However, the PC sensor system remains limited; as its reflection spectrum depends on the light incidence angle and position of the beam onto the sensor, making noise a much larger factor. Furthermore, biofluids surrounding the sensor results in absorption of incident light, decreasing light intensity, and resulting in poor sensitivity and accuracy. However, the device concepts developed in this sensor may introduce alternatives to electrical approaches in implantable sensing devices. Rather than using another optomechanical sensor to measure ICP, Zhang et al. [207] developed an ICP sensor using a silk fibroin-based hydrogel (CSFH) as a mechanoreceptor (Fig. 10g). Silk proteins were selected due to its ease in incorporating a variety of dopants, to impart properties as desired. In this case, a silk fibroin hydrogel was doped with carbon nanotubes to increase its conductivity for use as an implantable pressure and strain sensor, and with papain to allow for light-triggered degradation at will. The CSFH pressure sensor was made by sandwiching CSFH between aluminum-coated silk film electrodes and then implanted in Sprague–Dawley rats’ intracranial space. The CSFH sensor was able to detect differences in ICP during the normal state, during epilepsy, and after euthanasia, demonstrating its efficacy for real-time ICP monitoring. Rather than using hydrogels in an ICP sensor, Park et al. [166] developed hydrogel hybrid probes to record electrophysiological signals associated with optical stimulation (Fig. 10h). Using a soft PAAm-Alg hydrogel matrix, the hybrid probes can minimize the chemo-mechanical mismatch between the probes and surrounding tissues. The PAAm-Alg hydrogel has a bending stiffness that is dependent on its level of hydration; the dehydrated hydrogel has a high bending stiffness suitable for implantation, while post-implantation the hydrogel quickly absorbs water from surrounding tissue, allowing it to mechanically match surrounding tissue. After implantation, the signal-to-noise ratio (SNR) increased gradually over the first four weeks post-implantation and plateaued for an additional five months. This indicates the ability of the hydrogel hybrid probes to mechanically match to the surrounding tissue, and the efficacy of the implanted probes to measure the brain's long-term activity. Another electrophysiological signal sensor had previously been developed by Yu et al. [198]; however, instead of a hydrogel sensor, bioresorbable silicon electrodes were used instead to measure in vivo electrophysiological signals in adult rat animal models (Fig. 10i). The sensor used doped Si NM as the active sensing region, with a film of SiO_2_ as an encapsulating layer, and a resorbable PLGA substrate. The cortically implanted electrodes were able to measure neural activity comparably to a control electrode, indicating the sensor's ability to reliably measure the brain's electrophysiological and pathological activity. Both electrocorticography (ECoG) system and intracranial pressure were incorporated onto a single fully biodegradable sensing platform [203] (Fig. 10j). While a biodegradable composite material of poly(l-lactide) and polycaprolactone (PLLA/PCL) has shown promise for use in implantable devices, with a slow rate of degradation, high flexibility and malleability, and transparency and colorlessness for optical sensing, it has never been demonstrated in an ECoG system or other bioresorbable sensing platforms. During in vivo tests, the ECoG sensor was implanted onto the primary somatosensory cortex. The sensor could differentiate between different stages in rest, and between induced absence and grand mal seizures, displaying excellent sensitivity with high SNR while measuring neural signals, with a functional lifetime of 5 days, enabled by its use of PLLA/PCL encapsulating material.

### Intraocular Pressure Monitoring

**Fig. 11:**
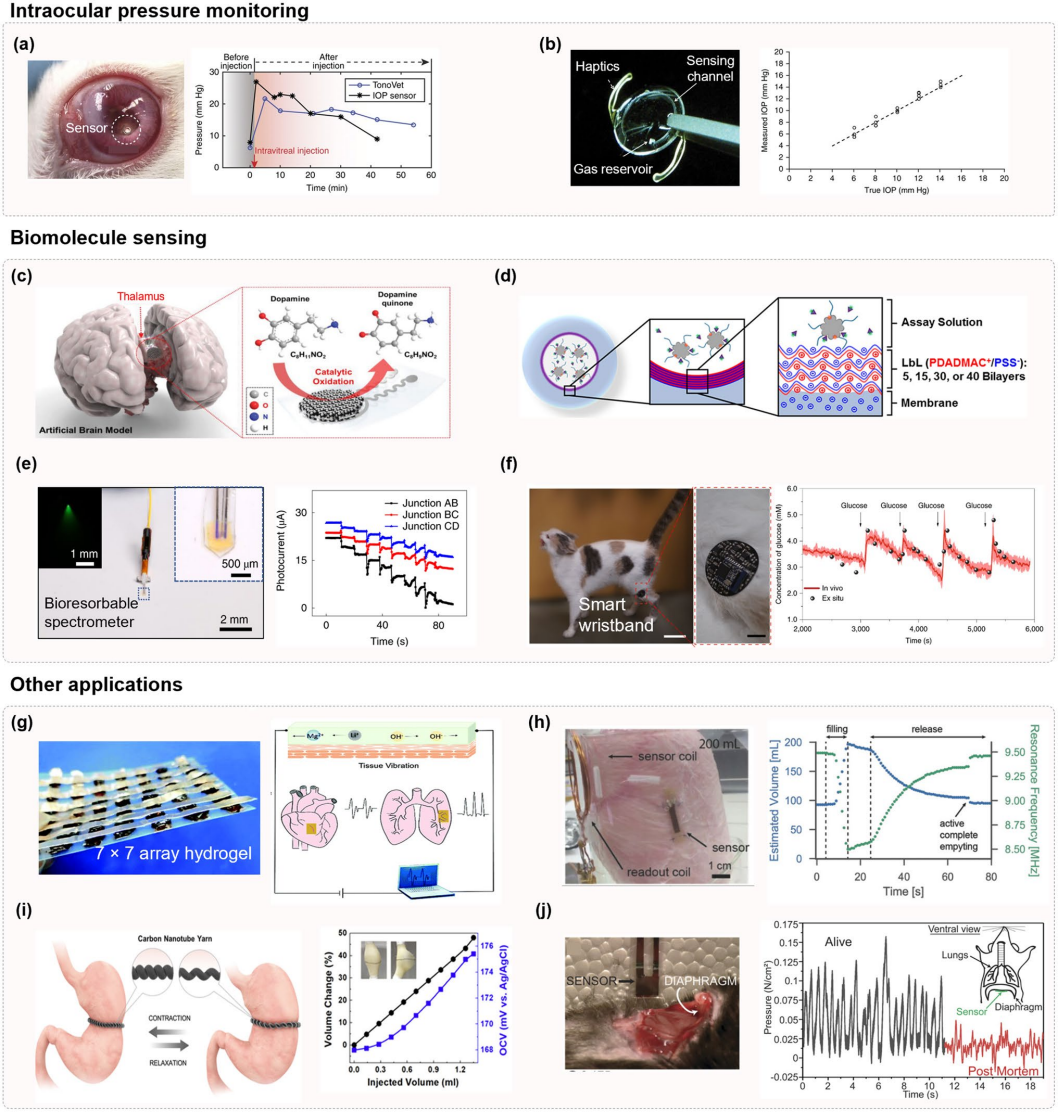
Advanced wireless, batteryless, implantable electronics system for in vivo physiological signal monitoring applications: Intraocular pressure monitoring (**a**, **b**), Biomolecule sensing (**c**–**f**), other applications (**g**–**j**). **a** Reproduced with permission [225] Copyright 2017, Springer Nature. **b** Reproduced with permission [224] Copyright 2014, Springer Nature. **c** Reproduced with permission [205] Copyright 2018, Wiley-VCH. **d** Reproduced with permission [226] Copyright 2018, ACS. **e** Reproduced with permission [227] Copyright 2019, Springer Nature. **f** Reproduced with permission [152] Copyright 2020, Springer Nature. **g** Reproduced with permission [14] Copyright 2020, RSC. **h** Reproduced with permission [15] Copyright 2018, Wiley-VCH. **i** Reproduced with permission [13] Copyright 2019, ACS. **j** Reproduced with permission [228] Copyright 2018, PNAS

The measurement of intraocular pressure (IOP) is a key parameter for the treatment of glaucoma [224]. However, IOP is often only measured infrequently a few times a year, making it difficult to obtain an accurate picture of a patient’s IOP history. In addition, most methods of IOP measurement, including the standard tonometry, only indirectly measure IOP, and are hindered by complex operation requirements and individual variations in corneal biomechanics. Previous implantable sensors have been developed, but are often large in the millimeter-to-centimeter range, resulting in damage to surrounding tissue. As a result, Lee et al. [225] developed a microscale IOP sensor, consisting of a pressure-sensitive optical resonant cavity made with a SiN membrane and Si mirror (Fig. 11a). At a given IOP, the sensor’s membrane deforms inwards and decreases the gap in the cavity, resulting in a unique resonance spectral signature for each IOP. A gold nanodot array was added to the SiN membrane to optimize the reflectivity of the SiN membrane, matching it with the reflectivity of the reflective bottom Si mirror to maximize the optical resonance amplitude. Both benchtop and in vivo testing of the IOP nanodot sensor found consistency in the readings compared to IOP measurements measured concurrently with a commercial rebound tonometer for up to 4.5 months, demonstrating its efficacy as an IOP sensor.

A similar device was developed by Narasimhan et al. [229] However, instead of using a gold nanodot array to enhance the optical signals, anti-reflective black silicon (b-Si) was used to cover inactive portions of the sensor to absorb and scatter incoming light. This enabled the suppression of background noise and improvement in the sensor’s signal-to-noise ratio. Using benchtop testing, the presence of b-Si was found to reduce background noise by more than a factor of ten when compared to a control sensor without b-Si, reducing the root mean square error and peak-to-peak variation by 70% and 75%, respectively. Rather than using a single optomechanical cavity, Siddique et al. developed a novel optomechanical nanophotonic sensor using flexible 3D hybrid photonic crystal (HPC) to measure IOP [230]. The HPC consists of sponge-like spherical air voids in PDMS or medical-grade silicon. As intraocular pressure changes, the nanoscale cavities within the HPC deflect, resulting in a blueshifted resonance, the location of which can be identified with a commercial mini-spectrometer to determine IOP. The sensitivity of the sensor depends on the Young’s modulus of the material it is fabricated from; from benchtop testing, it was found that the sensitivity of the softer PDMS sensor (0.4 nm mm^−1^ Hg) is greater than that of the medical-grade silicon (0.1 nm mm^−1^ Hg). However, both sensors displayed good linearity at the physiological IOP range (0–40 mm Hg) compared to a digital pressure gauge, with errors lower than that of a commercial tonometer across six months of implantation. In Araci et al. [224], another mode of ICP measurement was developed; in this study, a microfluidic device was developed for self-monitoring of IOP (Fig. 11b). The sensor consisted of an airtight microfluidic channel incorporated into an intraocular lens (IOL) used in cataract surgery. The microfluidic channel was open to intraocular aqueous liquid on one end, and a gas reservoir on the other. IOP can be assessed by visual inspection of the interface between the gas and aqueous intraocular liquid using a camera; a decrease in IOP shifts the interface toward the opening, while an increase in IOP shifts the interface further into the microfluidic device. Both benchtop and in vivo testing of the sensor found excellent sensitivity to pressure changes and reproducibility; this offers a promising alternative for glaucoma monitoring after hospital discharge. As demonstrated by the previously discussed sensors, many intraocular sensors use optical methods for sensing of intraocular pressure. While glaucoma is often linked to high IOP, a link between glaucoma and presbyopia, farsightedness as a result of loss of elasticity in the eye has been hypothesized [231]. However, few sensors have been developed to measure strains in the eye. As a result, Fernandes et al. have developed a novel optical strain sensor based on near-wavelength high-contrast grating arrays fabricated from etched SiO_2_ on an elastomeric PDMS substrate. By combining nanophotonic grating structures with an elastomeric material, small strains can be observed through visual inspection alone as a measurable change in color [231].

### Biomolecule Sensing

Biomolecule sensors are often used to monitor the presence of dopamine. However, while platinum (Pt) nanoparticles have been used in the past as catalyst, it is not bioresorbable [205] (Fig. 11c). In Kim et al., silicon nanomembrane electrodes were coated in cheaper, bioresorbable iron-based nanoparticles as an alternative to non-transient Pt nanoparticles. This substitution imparted equivalent catalytic functionality for the selective detection of dopamine while allowing the resulting dopamine sensor to be fully bioresorbable. To produce the sensor, Si NM electrodes coated in the iron-based NP were fabricated onto a flexible PCL substrate. The sensor was assessed by assessing the changes in conductance in the dopamine sensors during exposure to varying concentrations of DA. While negligible changes in current occurred when the electrodes were exposed to solutions containing neurotransmitters other than DA, observable changes were measured when the neurotransmitter solution contained 1 × 10^−6^ M of DA, highlighting the selective detection of DA by the sensor. While dopamine is frequently targeted by biomolecule sensors, continuous glucose monitoring (CGM) is another essential application, particularly in managing diabetes mellitus. Electrochemical CGM sensors have been developed; however, such sensors rely on placement of transducers and sensing platforms in direct contact with the biomolecule in question [226] (Fig. 11d). In response, Locke et al. developed a novel optical continuous glucose monitoring system using a competitive fluorescent binding assay that fully resides in subcutaneous tissue. The competitive binding glucose sensing assay was contained in a hollow cavity formed within a cylindrical hydrogel membrane. Across glucose concentrations of between 50 and 600 mg dL^−1^, an increasing Förster resonance energy transfer (FRET) response was observed, indicating the responsivity of the sensors to varying concentrations of glucose for short intervals of time. However, while bilayers were incorporated to minimize loss of the assay, further refinement to further reduce assay diffusion should still be conducted. Another optical sensor was developed for the biomolecule detection by Bai et al. [227] (Fig. 11e). However, rather than using a binding assay, a photonic device was developed to spectroscopically detect critical biochemical species along with physiological signals. The photonic device was made only of biodegradable materials, consisting of a doped Si NM photodetector, zinc electrodes, a PLGA substrate and bioresorbable optical fiber. However, while many other devices still require an invasive surgical procedure to place the device, Bai et al.’s device was shaped to resemble a hypodermic needle with dimensions of only around 600 µm wide and 160 µm thick, allowing the device to be placed through a minimally invasive injection process. By using a tuneable laser light source with the photonic device, a transmission spectrum could be generated to identify the presence of biomolecules such as glucose, albumin, melanin, and more. Another injectable device was developed by Wang et al. [152], who developed an electrochemical sensor to detect disease biomarkers in real time and in vivo (Fig. 11f). Rather than mimicking the shape of a hypodermic needle, Wang et al.’s device uses helical carbon nanotube fiber bundles to mimic the hierarchical structure of muscles to increase the flexibility of the electrochemical sensor and avoid mechanical mismatch once implanted. By incorporating different sensing components into single-ply sensing fibers (SSF), such as Pt nanoparticles to catalyze H_2_O_2_ dissociation, biomolecules can be detected with specificity; by twisting multiple fibers together, a multi-ply sensing fiber (MSF) capable of detecting multiple biomarkers was developed to detect H_2_O_2_ in tumors, and glucose and Ca^2+^ ions in the blood.

### Other Applications

Biomimetic structures, strong adhesion, and sufficiently conformal contact are desirable when attempting to measure organ motion [14, 15]. Pei et al. [14] suggested an unique approach to develop a novel hydrogel strain sensor, with a PDA–clay–PSBMA hydrogel material inspired by mussels that are wet adhesive, ion conductive, self-healing, and highly flexible (Fig. 11g). By coupling wireless transmitters, wireless transmission of the real-time sensing performance of the hydrogel-based sensor could be achieved. With its strong adhesion, the hydrogel could be used to seal and help repair injuries, and aid in postoperative monitoring. Furthermore, with close conformal contact, and high ionic conductivity, the mussel-inspired PDA–clay–PSBMA hydrogel sensor demonstrated high sensitivity with a gauge factor of 2 at high strains up to 200%. On top of organ motion, various implantable devices have been developed to monitor other organ behaviors, such as changes in bladder volume [15] (Fig. 11h). Passive wireless resonant sensors have been proposed for measurement of biosignals to avoid the use of active components; however, few passive sensor solutions have been suitable for high deformations greater than 20% that do not interfere with bladder filling. As a result, Stauffer et al. developed a soft, implantable bladder volume sensor with a continuous wireless readout system that avoids use of active components. The stretchable sensor consists of an implantable RLC circuit with a stretchable capacitor adhered to the bladder. The capacitor was made from gold-coated titanium dioxide nanowire (Au–TiO_2_ NW) embedded in an ultrasoft silicone elastomer matrix. The thickness of the composite was reduced to five to ten times thinner than the bladder wall to reduce mechanical influence on the bladder. Furthermore, the wireless readout system uses an external coil with a frequency sweep to detect the resonance frequency of the sensor. By using an operating resonance frequency between 1 and 20 MHz, the sensor could communicate through greater tissue depths compared to other wireless devices that use high MHz to GHz resonance frequencies. Just as the bladder can suffer from dysrhythmia so too can the stomach, where electrical signals controlling gastric contractions can be disrupted [13] (Fig. 11i). While implanted gastric stimulators can help restore gastric contractions, more effective gastric contraction monitoring is still required. As a result, Jang et al. developed a gastric deformation sensor that takes advantage of stretch-induced changes in capacitance of coiled carbon nanotube (CNT) to measure stomach contractions. The sensor consists of a coiled CNT yarn which acts as an energy harvester, using the capacitance changes of the yarn due to tensile stroke or torsional rotation to generate an electrical voltage. The CNT yarn could withstand 30% strain for 30,000 cycles at 1 Hz without change in functional properties, suggesting that the device could accurately assess frequency and amplitude of peristaltic stomach volume changes for an extended period. Rather than measuring organ strain, organ pressures can also be measured to assess the condition and behavior of organs [228] (Fig. 11j). Previous work has been conducted on bioresorbable force sensors; however, many such sensors still require complex clean room fabrication tools to be fabricated. To rectify this, Curry et al. used molybdenum, PLA, and PLLA to create a bioresorbable and piezoelectric PLLA pressure sensor that required only a simple fabrication process. The sensor consisted of two layers of PLLA sandwiched between two Mo electrodes and encapsulated in PLA. To fabricate the sensor, PLLA was annealed and stretched to impart PLLA’s piezoelectric properties. The Mo electrodes were affixed to the top and bottom of the PLLA film, and the assembly was then sandwiched between layers of PLA film. The PLA is then sealed by PLA glue and then using a commercial plastic sealer. The accuracy of the device was verified using a commercially available piezoelectric quartz force sensor, and the device was able to withstand 10,000 cycles of up to 1 MPa of force without loss of accuracy. Thus, Curry et al. demonstrated a PLLA pressure sensor that could accurately measure internal organ forces that required only a simple fabrication process compared to standard photolithography-assembled sensors.

## Conclusion and Outlook

**Fig. 12:**
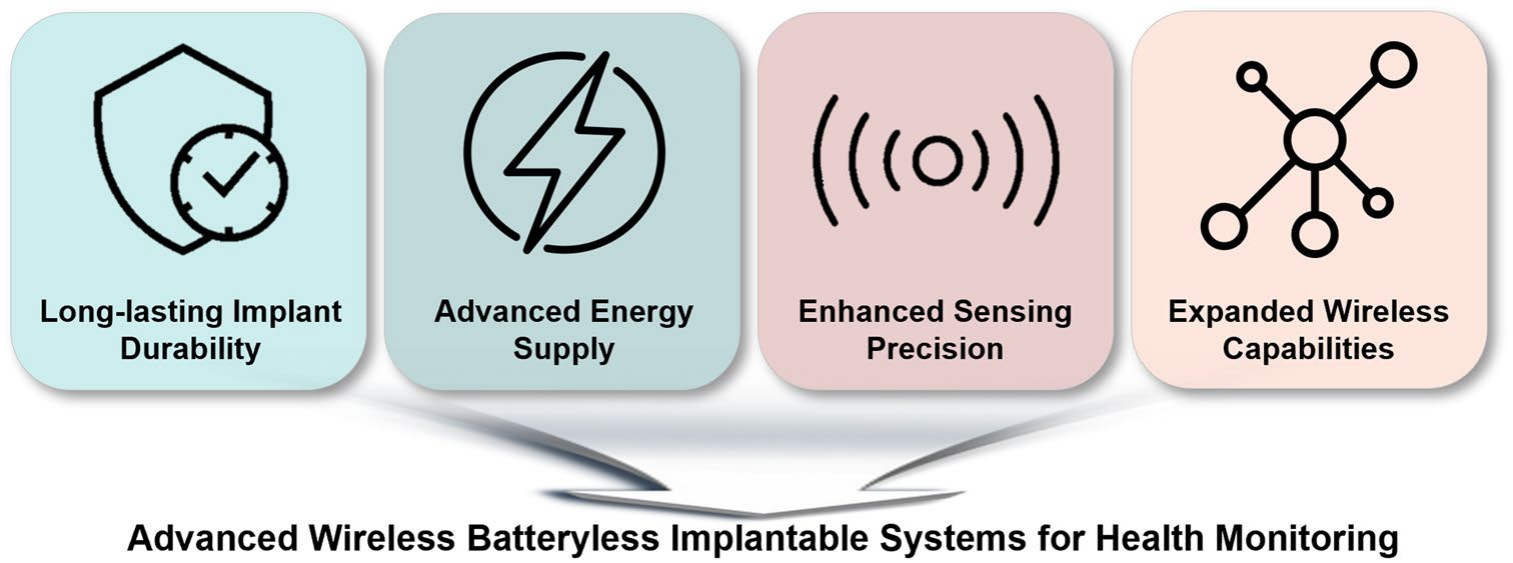
Illustrative summary highlighting key advancements in the wireless batteryless implantable systems for health monitoring

In line with the increasing demand for personalized health care and remote continuous patient monitoring, wireless implantable sensors and electronics are expected to gain widespread popularity. Moreover, the convergence of multi-disciplinary research efforts, including material sciences, wireless communication, wireless power transfer, biomedical engineering, electronics, and health care, has accelerated the development of wireless implantable electronics systems. Although numerous challenges need to be overcome during the design process of implantable devices, they can offer unprecedented insights into various processes that occur inside an organism with unmatched timing resolution. Consequently, this unique implantable platform holds great promise for investigating physiological phenomena, facilitating medical diagnoses, and controlling drug delivery. However, while recent breakthroughs have propelled the development of implantable biosensors, numerous challenges remain to be addressed. Due to the devices being inserted inside the organism, they will be in constant contact with different biological tissues. Recently advanced materials do not incite major immunological or inflammatory responses during the implantation, offering proper implant shape and physical properties needed to match the surrounding tissue to minimize damage. Another interesting development in materials has been the advent of bioresorbable materials. Additional surgical procedures will be needed to remove the implanted device, which can expose the patient to unwanted risks. However, keeping the device can also lead to a more drastic immunological response. In such a case, the implantable system with biodegradable features offers excellent potential for implants that the body will safely absorb after a predetermined time without additional surgery. Even if the device is needed for long-term monitoring, unnecessary surgeries may still be needed if it does not have a wireless data retrieval procedure or contains batteries that need replacing. Effective retrieval of data from the device is essential for optimal utilization of an implantable sensor. But wires are prone to infections, and having a recoverable internal memory would subject the patient to an additional medical procedure. Therefore, wireless techniques are the best candidates for this task. Recent advances in passive wireless devices show advantages due to their simplicity in device implantation, energy supply, and data acquisition. In the case of active wireless systems, while data transmission from the device to the outside system may require more power, it facilitates the execution of more complex behaviors. The most convenient way to power the devices implanted inside the body is to use energy harvesters, which will not require any external input to provide a constant and reliable energy source. Although they offer a more reliable power source when compared to WPT, the latter can offer a more generous power budget. Lastly, we emphasize several significant challenges and corresponding research prospects (Fig. 12). First, some implants must remain with the patient for the rest of their lives. Therefore, these sensors must be designed to endure long-term stress without losing their functionality. Especially in the case of soft devices, the mechanical integrity of the sensors needs to be retained even after millions of stretching cycles. Moreover, it is also necessary for these devices to keep their biocompatibility for as long as they are implanted. Thus, the integrity of the encapsulation layer and the design at the biointerface should be designed to reach or even exceed human lifespans. Therefore, future investigation needs to be undertaken about long-term in vivo trials of soft implantable devices, especially those that incorporate stretchable mechanics. Second, reliable power supply strategies still need to be developed to monitor physiological indicators using implantable devices continuously. Improvements to existing energy harvesting techniques, especially those that can convert energy inside the body, are needed to power more complex devices. Moreover, when harvesting energy from outside the body, it is necessary to create strategies to mitigate the effects of changes in the environment on the power budget of the implants. Utilizing stable wireless powering techniques will ultimately be required to expand the possibility of practical approaches. Third, the sensing capabilities of implants and the accuracy of the retrieved data also need to be enhanced. An increase in the reliability, quality, and volume of the data acquired by this class of biosensors would not only provide more precise diagnostics. Still, it would also allow new opportunities to use artificial intelligence models. The use of machine learning would offer a multi-disciplinary direction for medical diagnoses, such as personalized health care and research and development in biomedical engineering. For instance, interactions of new drugs and treatments with the target tissue could be better understood and monitored with the use of such devices in both animal and clinical trials, allowing for improvements in the current testing procedures. Finally, there is an opportunity for the improvement of the range and bandwidth of wireless devices, as well as a capability to increase the computational capabilities available with the advent of higher energy-efficient electronics; the increase in bandwidth of the wireless communication scheme would allow for the enhancement of the timing resolution of the implanted devices. Moreover, the increased computing power included in the implants would allow for on-device preprocessing of the data, which would be especially beneficial in solving one of the biggest concerns with implantable devices currently: securing the information. All the information exchanged between the biosensor and the external world must be encrypted to obtain implants safe for everyday use. Wireless, batteryless implantable biosensors will perform an important role in the future of healthcare and biomedical research. In addition to the advantage of this unique system, this good technique will contribute to synergetic multi-disciplinary achievement for medical approaches and research and development in biomedical engineering.
